# Rab40–Cullin5 complex regulates EPLIN and actin cytoskeleton dynamics during cell migration

**DOI:** 10.1083/jcb.202008060

**Published:** 2021-05-17

**Authors:** Erik S. Linklater, Emily D. Duncan, Ke-Jun Han, Algirdas Kaupinis, Mindaugas Valius, Traci R. Lyons, Rytis Prekeris

**Affiliations:** 1 Department of Cell and Developmental Biology, School of Medicine, University of Colorado Anschutz Medical Campus, Aurora, CO; 2 Proteomics Center, Institute of Biochemistry, Vilnius University Life Sciences Center, Vilnius, Lithuania; 3 Division of Medical Oncology, Department of Medicine, School of Medicine, University of Colorado Anschutz Medical Campus, Aurora, CO; 4 University of Colorado Cancer Center, Young Women’s Breast Cancer Translational Program, Aurora, CO

## Abstract

Rab40b is a SOCS box–containing protein that regulates the secretion of MMPs to facilitate extracellular matrix remodeling during cell migration. Here, we show that Rab40b interacts with Cullin5 via the Rab40b SOCS domain. We demonstrate that loss of Rab40b–Cullin5 binding decreases cell motility and invasive potential and show that defective cell migration and invasion stem from alteration to the actin cytoskeleton, leading to decreased invadopodia formation, decreased actin dynamics at the leading edge, and an increase in stress fibers. We also show that these stress fibers anchor at less dynamic, more stable focal adhesions. Mechanistically, changes in the cytoskeleton and focal adhesion dynamics are mediated in part by EPLIN, which we demonstrate to be a binding partner of Rab40b and a target for Rab40b–Cullin5-dependent localized ubiquitylation and degradation. Thus, we propose a model where Rab40b–Cullin5-dependent ubiquitylation regulates EPLIN localization to promote cell migration and invasion by altering focal adhesion and cytoskeletal dynamics.

## Teaser

This study shows that Rab40b interacts with Cullin5 and that loss of Rab40b–Cullin5 binding affects stress fiber formation and focal adhesion dynamics. Mechanistically, these effects are mediated in part by EPLIN, which is a target for Rab40b–Cullin5-dependent localized ubiquitylation and degradation.

## Introduction

Cell migration is a complex and highly regulated process that involves coordinated changes in signaling, membrane trafficking, and cytoskeleton dynamics. Consequently, during epithelial-to-mesenchymal transition in development or carcinogenesis, cells undergo an extensive shift in genetic and posttranslational programming to promote cellular pathways important for migration, such as the loss of cell–cell adhesion and enhanced localized dynamics of both the actin cytoskeleton and focal adhesions (FAs; Lamouille et al., 2014; Dongre and Weinberg, 2019). Additionally, it is well established that ECM degradation and remodeling play a key role in mediating cell migration during development and cancer metastasis. ECM remodeling is facilitated via the delivery and secretion of matrix metalloproteinases (MMPs) to the leading edge of invasive cells (Brooks et al., 1996; Chen and Wang, 1999; Kessenbrock et al., 2010; Shay et al., 2015; Jacob and Prekeris, 2015).

While the cellular machinery mediating this targeted release remains to be fully defined, it is clear that MMPs are released at cellular extensions known as migratory pseudopods or podosomes in normal cells or invadopodia in cancer cells (Chen, 1989; Murphy and Courtneidge, 2011). Regardless of the context, these cellular extensions are typically formed and extended via localized polymerization of the actin cytoskeleton and occur with the coordinated assembly/disassembly of FA sites (Badowski et al., 2008; Kolli-Bouhafs et al., 2014; Petropoulos et al., 2016). Thus, the key to cell migration through the ECM is the coordination between actin polymerization, FA assembly/disassembly, and targeted secretion of MMPs. How all these processes are integrated and regulated during cell migration remains to be fully understood and is a main focus of this study.

Since MMP targeting to invadopodia is one of the key events during cancer cell migration, we and others aim to identify regulators of this process. Rab GTPases are known master regulators of targeted vesicle transport and cargo secretion (Stenmark, 2009). Accordingly, roles for various Rabs in the delivery of MT1-MMP (also known as MMP14), including Rab5a, Rab8, Rab14, and Rab27a, have been established (Bravo-Cordero et al., 2007; Wiesner et al., 2013; Hoshino et al., 2013). Work from our laboratory specifically identified Rab40b as an important regulator of MMP2/9 secretion and ECM remodeling during breast cancer cell invasion. Relatedly, we have also demonstrated that Tks5, a known invadopodia regulatory protein, is an effector protein for Rab40b (Jacob et al., 2013; Jacob et al., 2016). Additional work from other laboratories has further corroborated an emerging role for Rab40b in cell migration and cancer progression (Li et al., 2015; Liu et al., 2018; Zacharias et al., 2018; Myat et al., 2019).

**Figure 1.**
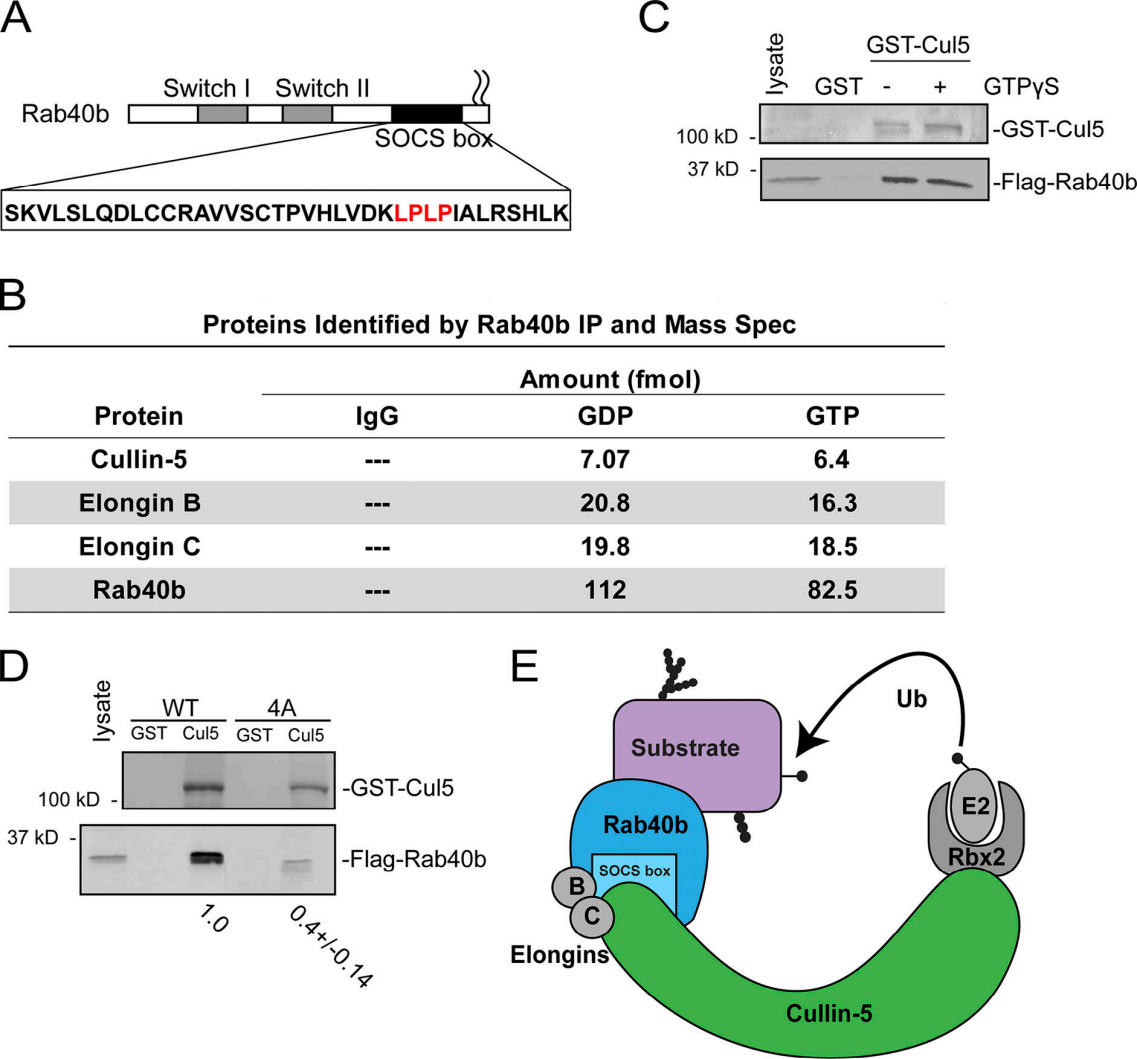
**Rab40b binds to Cullin5 in SOCS-dependent and GTP-independent manner.** **(A)** Schematic diagram of Rab40b. **(B)** List of proteins identified in both GDP- and GTPγS-bound FLAG-Rab40b immunoprecipitates from MDA-MB-231 lysate followed by mass spectroscopy analysis. **(C)** FLAG-Rab40b binding to GST-Cullin5 as analyzed by glutathione bead pull-down assay. Top panel: Coomassie staining. Bottom panel: WB with anti-FLAG. **(D)** FLAG-Rab40b (WT and 4A mutants) binding to GST-Cullin5 as analyzed by glutathione bead pull-down assay followed by WB with anti-FLAG (bottom blot) and Coomassie stain (top blot). Numbers shown are the average densitometry analysis derived from three independent experiments, standardized to WT FLAG-Rab40b. WT::4A, P < 0.001. **(E)** Model showing CRL5 E3 ligase complex partners. Ub, ubiquitin.

The Rab40 subfamily contains four closely related isoforms, Rab40a, Rab40al, Rab40b, and Rab40c, with Rab40a and Rab40al being expressed only in simian primates. This subfamily is unique among the Rab GTPases due to the presence of a C-terminal suppressor of cytokine signaling (SOCS) box motif (Fig. 1 A; Kile et al., 2002), which allows binding to members of the Cullin family of proteins (Kamura et al., 1998; Linossi and Nicholson, 2012). Cullins are the central component of the largest class of E3 ubiquitin ligases, referred to as Cullin-RING ligases (CRLs; Petroski and Deshaies, 2005; Gao et al., 2020).

Within the Cullin5-specific CRL (CRL5), Cullin5 acts as a scaffold protein that recruits RING box protein Rbx2, the Elongin B/C complex, and a SOCS box–containing adaptor protein. Together, this CRL5 complex mediates ubiquitylation of SOCS-bound substrate molecules. Cullin–SOCS complexes, first identified as scaffolds for JAK/STAT signaling (Starr et al., 1997; Endo et al., 1997; Naka et al., 1997), regulate a wide range of substrate proteins, including VHL (Kamura et al., 2004), DAB1 (Feng et al., 2007), and p130Cas (Feng et al., 2007). The canonical SOCS-containing family of 8 proteins has steadily been expanded to >40 (Okumura et al., 2016), including the Rab40 subfamily. Both Rab40a and Rab40c complex with Cullin5 to ubiquitylate Pak4 (Dart et al., 2015) and Rack1 (Day et al., 2018), respectively, ultimately resulting in their proteasomal degradation. However, little is known about the function of the Rab40b in this context, particularly in the setting of cell migration, invadopodia formation, and ECM remodeling. Furthermore, it remains unclear how much functional redundancy exists among the Rab40 isoforms.

We hypothesized that the Rab40b SOCS box would allow interaction with the CRL5 complex and contribute to coordination of MMP secretion by inducing changes in actin and FA dynamics. Consistent with this hypothesis, we demonstrate that Rab40b binds to Cullin5 in a SOCS box–dependent fashion and that formation of this CRL5 complex is required for chemotactic migration and breast cancer cell invasion. We also show that this Rab40b–Cullin5 complex influences multiple aspects of cell migration, including FA localization and dynamics, stress fiber induction, formation of invadopodia, and changes in lamellipodia dynamics. In this study, we completed an unbiased proteomics screen to identify Rab40b–Cullin5-specific substrate proteins. One of the proteins identified was EPLIN (epithelial protein lost in neoplasia; encoded by the LIMA1 gene), a tumor suppressor known to inhibit cell migration and epithelial-to-mesenchymal transition (Jiang et al., 2008; Sanders et al., 2011; Zhang et al., 2011; Maul and Chang, 1999; Liu et al., 2016). Here, we demonstrate that preventing Rab40b–Cullin5 binding increases total cellular EPLIN levels in breast cancer cells and causes its redistribution to stress fibers and the leading edge of migratory lamellipodia. Finally, we show that increased cellular EPLIN contributes to changes in FA and actin dynamics.

## Results

### Rab40b binds to Cullin5 in a SOCS-dependent and GTP-independent manner

**Figure S1.**
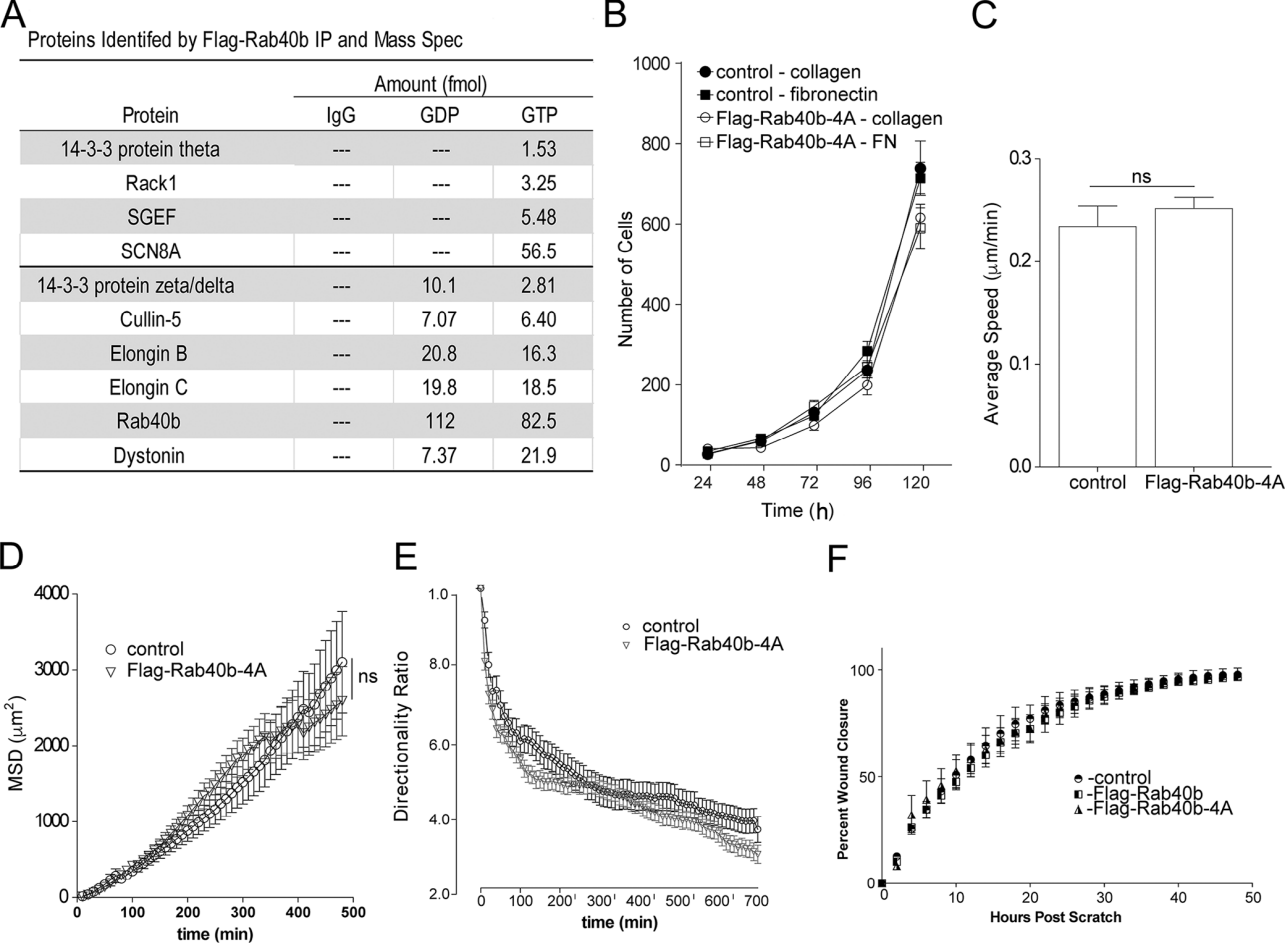
**Cell migration analysis. (A)** Table listing other proteins (in addition to Fig. 1) identified by mass spectroscopy analysis from FLAG-Rab40b pull-down. **(B)** Rates of cell proliferation of control and FLAG-Rab40b-4A cells on either collagen or fibronectin (FN). Data represent the means and SEM derived from three different experiments. **(C)** Average speed calculated from time-lapse analysis of parental (control) and FLAG-Rab40b-4A cells (also see Videos 1 and 2). Control, 0.24 µm/min ± 0.0 SEM, *n* = 51 cells; FLAG-Rab40b-4A, 0.25 µm/min ± 0.01 SEM, *n* = 121. **(D)** MSD calculated from time-lapse analysis of parental (control) and FLAG-Rab40b-4A cells (also see Videos 1 and 2). Data represent means and SEM derived from *n* = 39 control cells and *n* = 79 FLAG-Rab40b-4A cells. P = 0.75. For power-law equation fitting to MSD(Δt) = C*Δt^α^, where the exponent α is indicative of type of movement: control, MSD(Δt) = 0.8337Δt^1.3212^, α = 1.3212; FLAG-Rab40b-4A, MSD(Δt) = 0.6652Δt^1.3743^, α = 1.3743. **(E)** Directionality ratios control and FLAG-Rab40b-4A cells were calculated from time-lapse analysis (also see Videos 1 and 2). Only cells that remained in frame for the duration of the entire time-lapse experiment were analyzed. Directionality at the last time point is 0.38 ± 0.03 SEM for control cells and 0.29 ± 0.02 SEM for FLAG-Rab40b-4A cells. P < 0.05. **(F)** Migration analysis of control, FLAG-Rab40b, and FLAG-Rab40b-4A cells by scratch assay. Right: Representative images of cells at various time points. Blue boxes designate computer generated boundaries of original scratch border. Left: Quantification of three separate runs, with at least five wells per condition per run.

Identification of the full complement of Rab40b-interacting partners is necessary to define how Rab40b contributes to both cell migration and other biological processes. To identify additional Rab40b-binding partners, we first performed a proteomics screen. Due to the lack of reliable commercial antibodies for Rab40b, we used a previously generated triple-negative breast cancer cell line MDA-MB-231 that stably expresses FLAG-Rab40b (Jacob et al., 2013). Lysates from these cells were incubated with anti-FLAG antibody–conjugated beads or control IgG beads. Because Rab GTPases cycle between inactive GDP-bound and active GTP-bound states, we also immunoprecipitated FLAG-Rab40b in the presence of GDP or the nonhydrolyzable GTP analogue GTPγS. Bound proteins were then analyzed by mass spectrometry. Cullin5 as well as CRL5 complex partners Elongin B and Elongin C were identified as putative Rab40b-interacting proteins (Fig. 1 B and Fig. S1 A). All three proteins bound relatively equally to GDP- or GTPγS-bound Rab40b, suggesting that binding of the CRL5 complex is independent of the nucleotide state of Rab40b. To confirm that Cullin5 binds to Rab40b, we next incubated lysates from MDA-MB-231 cells constitutively expressing FLAG-Rab40b with glutathione beads conjugated to GST only or purified recombinant GST-Cullin5. Consistent with our mass spectrometry results, FLAG-Rab40b interacted with GST-Cullin5 independent of its nucleotide state (Fig. 1 C).

It is well established that a highly conserved LPLP motif, named the Cul-Box, is found within the SOCS box and that this motif is necessary for the binding of Cullin complexes to canonical SOCS proteins (Okumura et al., 2016). This motif is also present in Rab40b (Fig. 1 A, in red). To determine whether this motif is important for the binding of Rab40b to Cullin5, we mutated the LPLP sequence to AAAA (FLAG-Rab40b-4A), which has been shown to abate the binding of Cullin5 to other SOCS-containing proteins (Kamura et al., 2004). Lysates from MDA-MB-231 cell lines stably expressing either WT FLAG-Rab40b or FLAG-Rab40b-4A were used to test the ability of Rab40b to bind to GST-Cullin5 using glutathione bead pull-down assays. As shown in Fig. 1 D, mutant FLAG-Rab40b-4A has significantly reduced binding to GST-Cullin5 compared with WT. Taken together, these results demonstrate that mammalian Rab40b binds to Cullin5 and Elongin B/C independent of its nucleotide state and that binding to Cullin5 is mediated by the Rab40b SOCS box (Fig. 1 E).

### Rab40b**–**Cullin5 regulates cell migration via cytoskeletal reorganization

**Figure 2.**
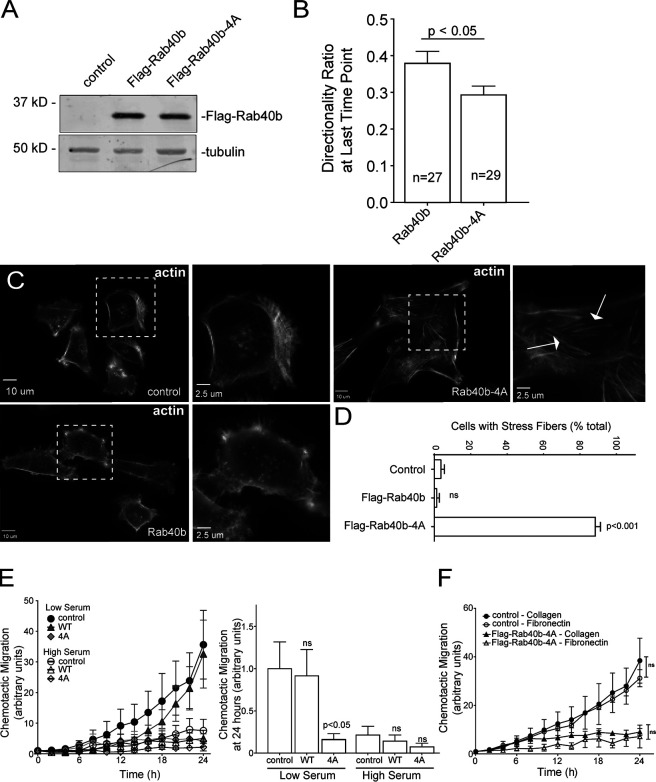
**Rab40b–Cullin5 affects individual cell migration.** **(A)** WB analysis of lysates from MDA-MB-231 cells stably expressing WT FLAG-Rab40b or FLAG-Rab40b-4A. **(B)** Directionality of migrating cells derived from time-lapse analysis of control or FLAG-Rab40b-4A cells (see Videos 1 and 2) for cells that remained in frame for the duration of the experiment. Data are shown as means and SEM. *n* is the number of cells analyzed. **(C and D)** Control, FLAG-Rab40b, and FLAG-Rab40b-4A cells were plated on collagen-coated coverslips and then fixed and stained with phalloidin–Alexa Fluor 594. Zoomed regions of interest highlight differences in cytoskeletal architecture, and arrows point to stress fibers. **(D)** Quantification of cells with prominent stress fibers. *n* ≥ 100 cells per condition. **(E)** Chemotactic assays of control, FLAG-Rab40b, or FLAG-Rab40b-4A cells on collagen coating, plated in either low- or high-serum conditions. Results are of three separate runs, with at least three wells per condition per run. Left panel: Grouped scatter plot of relative migration over the entire time course. Right panel: Bar graph showing relative migration at 24 h. **(F)** Chemotactic assays of control or FLAG-Rab40b-4A cells plated on either collagen or fibronectin. Results are of three separate runs, with at least three wells per condition per run.

Given that Cullin family members and their substrates are important regulators of cell migration (Chen et al., 2009; Simó and Cooper, 2013; Zhang et al., 2015; Teckchandani and Cooper, 2016), together with an emerging role for Rab40b in cell motility, we hypothesized that Rab40b and Cullin5 binding may be an important regulator of this process. Using MDA-MB-231 cells stably expressing either WT FLAG-Rab40b or the Cullin5 binding–deficient FLAG-Rab40b-4A mutant (Fig. 2 A), we first sought to assess potential differences in migration using a scratch wound assay. Somewhat surprisingly, expression of either WT or mutant Rab40b-4A had no discernable effect on cell migration (Fig. S1 F). Analysis of individual cells can often reveal changes in migration dynamics that can be missed using population migration assays, such as the scratch wound assay. Thus, we next sought to look for differences in individual cell movement. Control or FLAG-Rab40b-4A–expressing cells were plated on collagen-coated dishes, placed in full medium, stained with SiR-actin and DAPI, and imaged once every 10 min for up to 10 h and individually tracked (Videos 1 and 2). Generally, FLAG-Rab40b-4A–expressing cells were capable of moving in similar fashion to control cells, suggesting that disrupting Rab40b and Cullin5 interaction does not inhibit the cells’ ability to move. Tracking of individual cells allowed for quantification of various properties of cell migration, such as velocity, directionality, and persistence. Mean squared displacement (MSD), which quantifies the randomness of movement by assessing the amount of space explored by a particle within a system, showed no significant difference between control and FLAG-Rab40b-4A cells. When the MSD data are fit to the power-law function MSD(Δt) = C*Δt^α^, wherein the exponent α is indicative of the type of motion observed, both control and Rab40b-4A cells exhibit similar values that are representative of superdiffusive motion (Fig. S1 D; Dieterich et al., 2008; Metzler et al., 2014). Additionally, FLAG-Rab40b-4A cells showed no significant difference in velocity compared with control MDA-MB-231 cells (Fig. S1 C). Finally, we sought to assess “straightness” of cell movement by determining the cell directionality ratio, a measure of the net displacement of a cell from its starting to final position compared with the total distance traveled. Interestingly, FLAG-Rab40b-4A–expressing cells exhibit a slight but significant decrease in directionality (Fig. 2 B and Fig. S1 E), a phenotype that would be difficult to ascertain during scratch wound assays.

**Video 1.** **MDA-MB-231 cells stained with SiR-actin (yellow) and DAPI (blue), plated on collagen-coated glass-bottom dishes, and imaged every 10 min for 10 h. **Playback rate, 0.25 frames per second.

**Video 2.** **FLAG-Rab40b-4A–expressing cells stained with SiR-actin (yellow) and DAPI (blue), plated on collagen-coated dishes, and imaged every 10 min for 10 h.** Playback rate, 0.25 frames per second.

General observations from our live-cell imaging suggested differences in the actin cytoskeleton structure and dynamics, which are known to play a vital role in cell migration and invasion. Since Cullin family members, including Cullin5, have been shown to exert effects on the actin cytoskeleton (Chen et al., 2009; Hudson and Cooley, 2010; Teckchandani et al., 2014; Dubiel et al., 2018), we next sought to assess changes to the cytoskeleton that result from diminished Rab40b–Cullin5 binding. As shown in Fig. 2, C and D, expression of FLAG-Rab40b-4A enhanced formation of ventral actin stress fibers compared with control cells or cells expressing WT FLAG-Rab40b. Stress fibers are contractile actoMyosin bundles that contribute to mechanical force generation to regulate cell contractility, adhesion, and motility (Pellegrin and Mellor, 2007). Thus, the inability of cells to properly reorient or maintain their actin cytoskeletal architecture could explain why FLAG-Rab40b-4A–expressing cells can still move but have difficulties sustaining directional movement.

### Rab40b**–**Cullin5 regulates chemotactic migration and cell invasion

Having observed defects in cell directionality and cytoskeletal makeup in FLAG-Rab40b-4A cells, we hypothesized that Rab40b and Cullin5 binding may govern more complex modes of cellular movement. A defining feature of SOCS family member proteins is the ability to regulate cellular responses to extracellular signaling cues, so we next assessed how the loss of Rab40b–Cullin5 binding impacted chemotactic migration. Serum-starved control, WT FLAG-Rab40b, and FLAG-Rab40b-4A cells were seeded in low serum–containing chambers and allowed to migrate to chambers containing complete medium as a chemoattractant. As shown in Fig. 2 E, FLAG-Rab40b-4A cells demonstrated a significant reduction in migration toward chemoattractant. As a control, we also plated cells in chambers containing high serum. In these conditions, both control and WT FLAG-Rab40b cells, in addition to FLAG-Rab40b-4A cells, exhibit a reduced ability to migrate toward serum. These results suggest that movement by control and FLAG-Rab40b cells in the low-serum condition is driven by chemotaxis and that FLAG-Rab40b-4A–expressing cells are deficient in this ability.

Extracellular substrates can alter integrin signaling and differentially effect cell migration on particular substrates (Huttenlocher and Horwitz, 2011), so we next asked if different surface substrates would affect chemotactic migration of FLAG-Rab40b-4A cells. Fig. 2 F demonstrates that reduced chemotactic migration of FLAG-Rab40b-4A cells occurs on either collagen or fibronectin, suggesting that the Rab40b–Cullin5 complex may be part of a core chemotactic migration machinery rather than mediating a response to specific ECM components. Cell proliferation was not affected in our mutant cell line (Fig. S1 B), suggesting that the differences we observe in chemotactic migration are not due to inherent differences in cell division.

**Figure 3.**
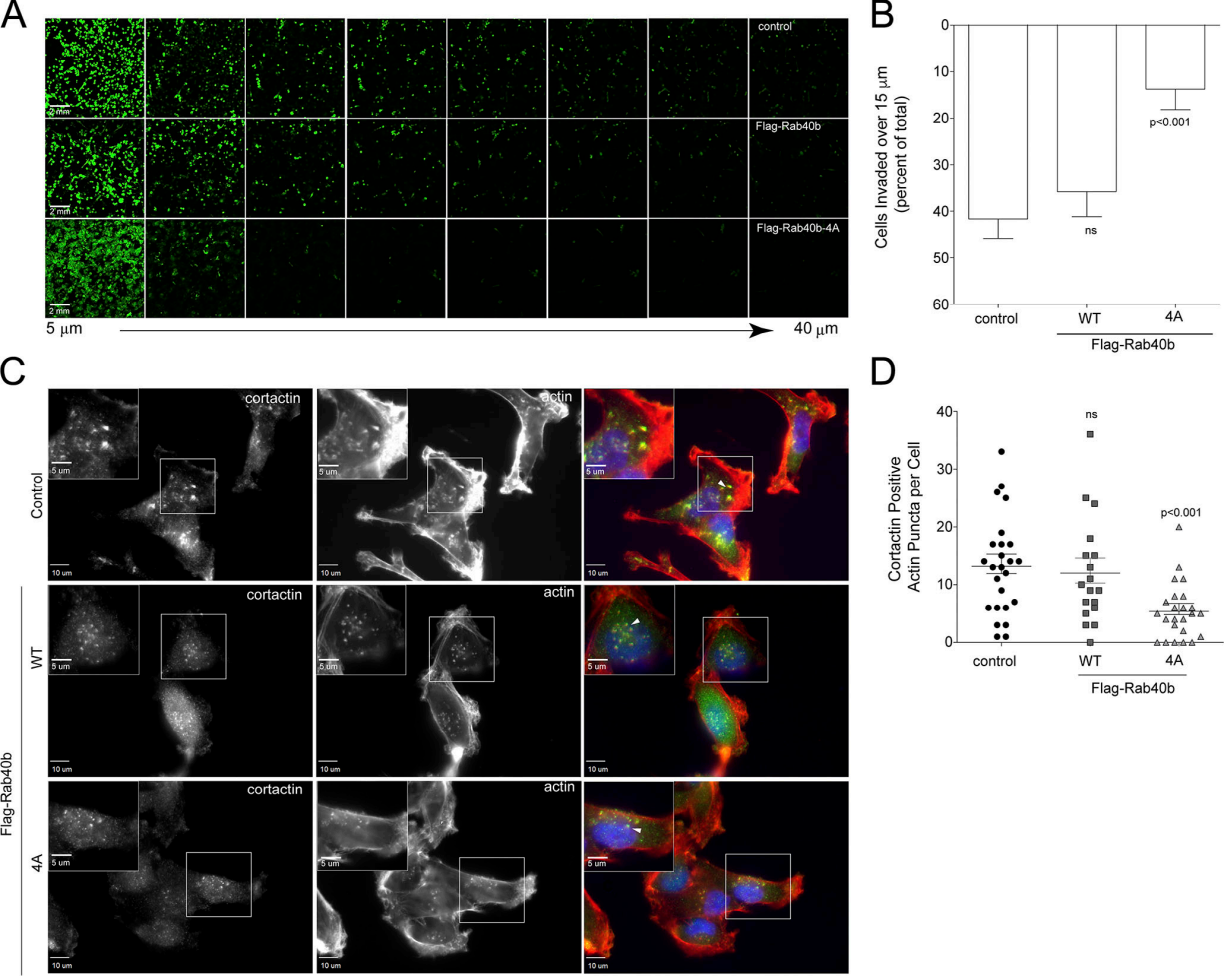
**Rab40b–Cullin5 regulates chemotactic migration and cell invasion.** **(A and B)** Matrigel invasion assay of control, FLAG-Rab40b, and FLAG-Rab40b-4A cells. Representative field-of-view images of Calcein-stained cells at 5-µm increments throughout the plug. **(B)** Quantification of cell migration through Matrigel plug. Results are of three separate runs, with at least three fields-of-view per condition per run. **(C and D)** Control, FLAG-Rab40b, or FLAG-Rab40b-4A cells plated on collagen-coated coverslips and then fixed and stained with phalloidin–Alexa Fluor 594 (red), cortactin (green), and DAPI (blue). Inset regions of interest highlight phalloidin/cortactin dual-positive puncta. Arrows point to actin/cortactin puncta. **(D)** Quantification of dual-positive actin/cortactin puncta. *n* ≥ 18 cells per condition.

Next, we sought to analyze how Rab40b and Cullin5 binding impacts cell migration in a 3D ECM environment. Using a modified inverted 3D Matrigel invasion assay, we found that while expressing WT FLAG-Rab40b had no significant impact on cell invasion compared with control cells, as expected (Jacob et al., 2016), cells expressing the FLAG-Rab40b-4A mutant had a significantly reduced ability to migrate through a 3D Matrigel matrix (Fig. 3, A and B). Degradation and remodeling of the ECM during cell migration is facilitated by protrusive actin structures called invadopodia. Our laboratory has previously demonstrated that Rab40b is required for the formation and function of invadopodia by regulating the secretion of MMP2/9. Given our current results showing a Rab40b–Cullin5-binding mutant induces changes to the actin cytoskeleton and impairs the cells’ ability to migrate through an extracellular environment, we hypothesized that the Rab40b–Cullin5 complex may function by coregulating MMP secretion with actin remodeling during invadopodia formation and extension. To test this hypothesis, we stained our cell lines with phalloidin–Alexa Fluor 594 along with an anti-cortactin antibody, an established marker of maturing invadopodia (Weaver et al., 2001; Clark et al., 2007), and then counted the number of cortactin-positive actin puncta per cell. Consistent with our previously published data (Jacob et al., 2013), overexpression of WT Rab40b had no effect on invadopodia number (Fig. 3, C and D). However, cells expressing FLAG-Rab40b-4A show a significant decrease in the number of invadopodia per cell (Fig. 3, C and D), suggesting that the ability of Rab40b to promote invadopodia formation depends on CRL5 binding. Since cortactin is also known to be present on late endosomes and lysosomes, we costained cells with cortactin and CD63, a well-established marker of the endolysosomal pathway. As shown in Fig. S3 A, expression of FLAG-Rab40b-4A had no effect on the number of cortactin- and CD63-positive organelles, suggesting that the Rab40b-4A mutant affects invadopodia formation rather than lysosomal. Previous work from our laboratory demonstrated the necessity of Rab40b in regulating secretion of MMP at invadopodia (Jacob et al., 2013), so we next assessed the level of secreted MMP2 and MMP9 from our cells. As shown in Fig. S3 E, the FLAG-Rab40b-4A cell line shows decreased secretion of MMP9 in a 2D gel zymography assay as compared with control and WT FLAG-Rab40b–expressing MDA-MB-231 cells. Surprisingly, we did not observe the decrease in MMP2 secretion. Thus, it remains unclear whether Rab40b binding to CRL5 is involved in regulating MMP secretion, since the FLAG-Rab40b-4A–induced decrease in MMP9 levels may be indirect result of changes in actin cytoskeleton and invadopodia formation.

### Rab40b**–**Cullin5 regulates localization and dynamics of FA sites

**Figure 4.**
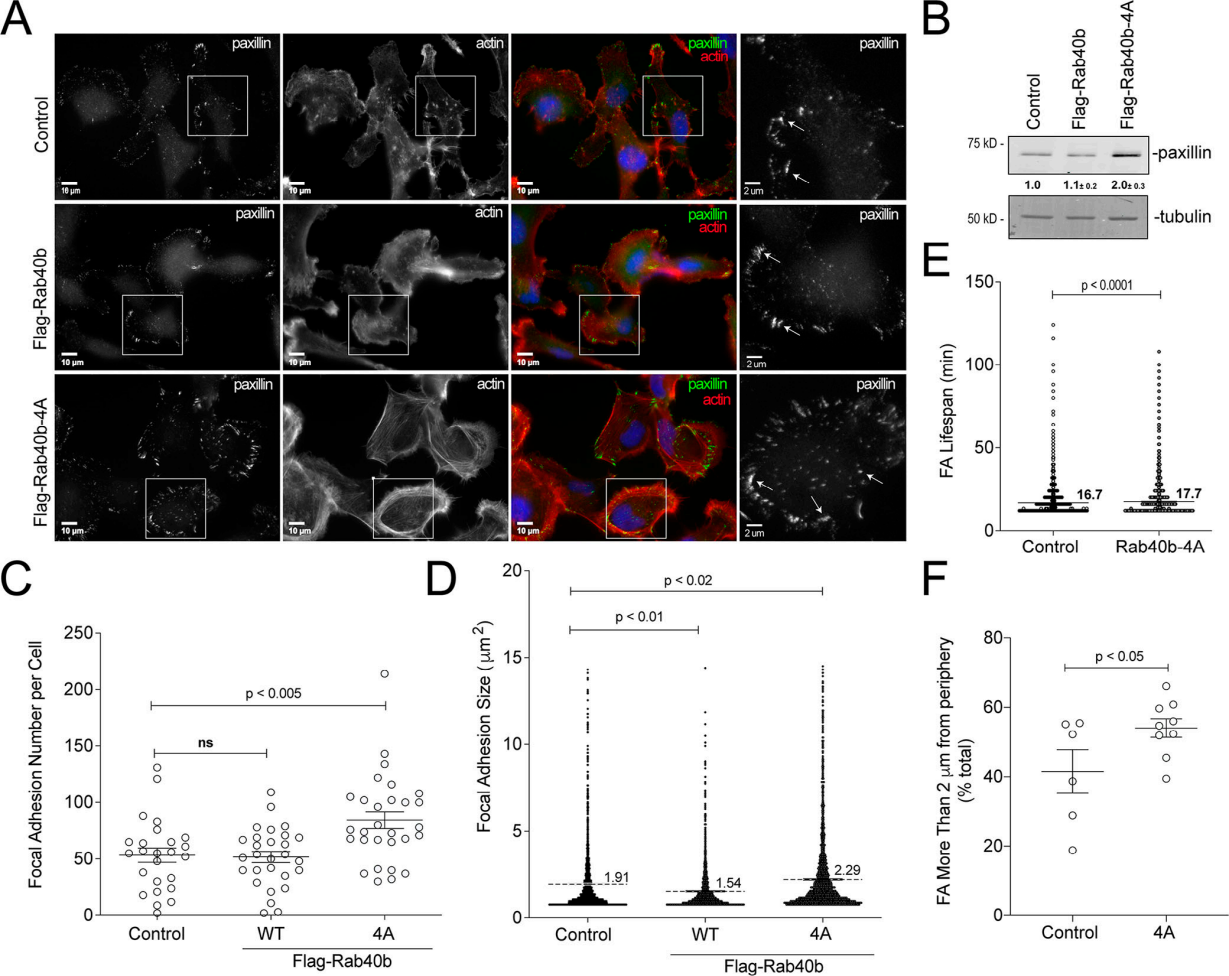
**Rab40b–Cullin5 regulates localization and dynamics of FA sites.** **(A)** Representative images of control, FLAG-Rab40b, and FLAG-Rab40b-4A MDA-MB-231 cells fixed and stained with phalloidin–Alexa Fluor 594 (red), anti-paxillin (green, FA marker), and DAPI (blue). Insets highlight anti-paxillin–stained regions of interest. **(B)** WB analysis of cell lysates using anti-paxillin (top blot) and anti-tubulin (bottom blot) antibodies. Numbers shown are densitometry analysis from at least three separate experiments relative to tubulin and standardized to control levels. Control::FLAG-Rab40b-4A, P < 0.05. **(C)** Quantification of number of FAs per cell for control, FLAG-Rab40b, and FLAG-Rab40b-4A cells. *n* ≥ 20 cells per condition. **(D)** Quantification of FA size in control, FLAG-Rab40b, and FLAG-Rab40b-4A cells. *n* = 2,479 total adhesions analyzed. **(E)** Quantification of FA average life span in control and FLAG-Rab40b-4A cells. Average life span of FAs was calculated from time-lapse images, with three cells per condition. **(F)** Quantification of percentage of FAs within 2 µm of cell border in control and FLAG-Rab40b-4A cells.

**Figure S2.**
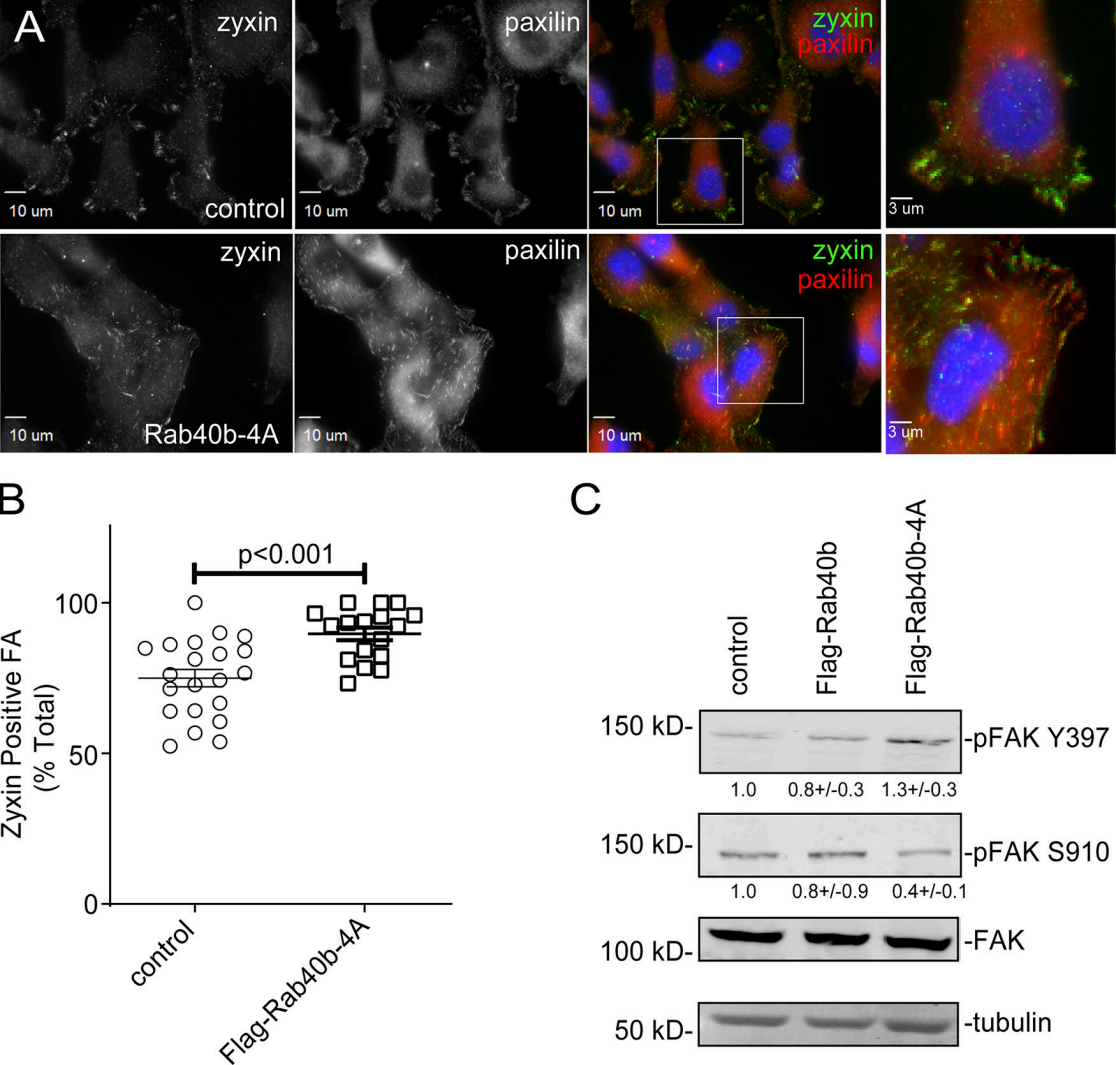
**FA maturation analysis. (A)** Images of control and FLAG-Rab40b-4A cells stained with zyxin (green) and paxillin (red). Boxes mark regions that are shown as higher-resolution images. **(B)** Quantification of percentage of zyxin-positive FAs in control and FLAG-Rab40b-4A cells. *n* ≥ 15 cells per cell line were analyzed. Control, 76.2 ± 2.9 SEM; FLAG-Rab40b-4A, 89.7 ± 2.1 SEM. **(C)** WB images of cell lysates blotted with anti-FAK, anti-Y397 pFAK, or anti-S910 pFAK antibodies. Numbers shown are the average densitometry analysis derived from three independent experiments relative to tubulin and standardized to control levels. Control::FLAG-Rab40b, S910 P = 0.05; Control::FLAG-Rab40b-4A, S910 P < 0.005, Y397 P < 0.05.

Stress fibers are known to connect to FAs and regulate FA dynamics (Burridge and Guilluy, 2016). Furthermore, FA dynamics has been shown to be regulated by Cullin5 (Teckchandani and Cooper, 2016). To assess whether the FLAG-Rab40b-4A–dependent increase in stress fibers was accompanied by an increase in FAs, we next stained MDA-MB-231 cells with antibodies against the FA marker paxillin. As shown in Fig. 4, A, C, and D, we see an increase in both number and size of FAs in the FLAG-Rab40b-4A mutant cells. Western blot (WB) analysis also shows an increase in paxillin protein levels in FLAG-Rab40b-4A cells compared with control and FLAG-Rab40b lines (Fig. 4 B). To determine whether the increase in FA number was due to an increase in their stability, we transfected either control or FLAG-Rab40b-4A cells with GFP-paxillin and SiR-actin and imaged them every 4 min for up to 7 h (Videos 3 and 4). Images were then uploaded to the FAAS server to assess FA life span. As shown in Fig. 4 E, FAs are longer lived in Rab40b-4A mutant cells. Additionally, Rab40b-4A mutants have a greater percentage of adhesions that are further from the cell boundary (Fig. 4 F), a result consistent with more stable, less dynamic adhesions (Legerstee et al., 2019). Consistent with this, FAs in FLAG-Rab40b-4A cells were more likely to be zyxin positive (Fig. S2, A and B), a marker for more mature FAs (Choi et al., 2008), again suggesting FAs in Flsg-Rab40b-4A–expressing cells are more stable than in their control counterparts.

**Video 3.** **MDA-MB-231 cells transfected with GFP-paxillin (green), stained with SiR-actin (red), plated on collagen-coated glass-bottom dishes, and imaged every 4 min for 280 min.** Playback rate, 0.25 frames per second.

**Video 4.** **FLAG-Rab40b-4A–expressing cells transfected with GFP-paxillin (green), stained with SiR-actin (red), plated on collagen-coated glass-bottom dishes, and imaged every 4 min for 280 min.** Playback rate, 0.25 frames per second.

FAK regulates FA formation and dynamics. Thus, we hypothesized that differences in FAK signaling may contribute to differences in FA dynamics in FLAG-Rab40b-4A cells. We assessed the level of two different FAK phosphorylation sites: Y397 and S910. Y397 is an autophosphorylation site that enables binding of Src and FAK activation and promotes FA stability (Hamadi et al., 2005; Horton et al., 2016). In contrast, S910 has been implicated in stimulating paxillin binding and turnover (Chu et al., 2011) and is necessary for invasive migration (Berg et al., 1988). As shown in Fig. S2 C, in the FLAG-Rab40b-4A cell line, we see an increase in phospho FAK (pFAK) Y397 and a decrease in pFAK S910 compared with control and WT-expressing cells, while total FAK remains constant. This result is consistent with the hypothesis that our observed increase in FA stability is due, in part, to differences in FAK signaling.

So far, our data demonstrate that expression of FLAG-Rab40b-4A mutant leads to changes in the size, number, and subcellular distribution of FAs. To test whether blocking Rab40b and Cullin5 binding also leads to changes in cell–ECM adhesion, we plated control and FLAG-Rab40b-4A cells on collagen coverslips and allowed them to adhere and spread for 90 min. Cells were then fixed and surface area of spreading visualized and analyzed by staining with phalloidin–Alexa Fluor 594. As shown in Fig. S5 A, FLAG-Rab40b-4A cells spread faster than control cells, indicating that the increase in FAs functions to adhere cells to the substrate.

### EPLIN is a Rab40b-binding protein that is ubiquitylated by the Rab40–Cullin5 complex

**Figure S3.**
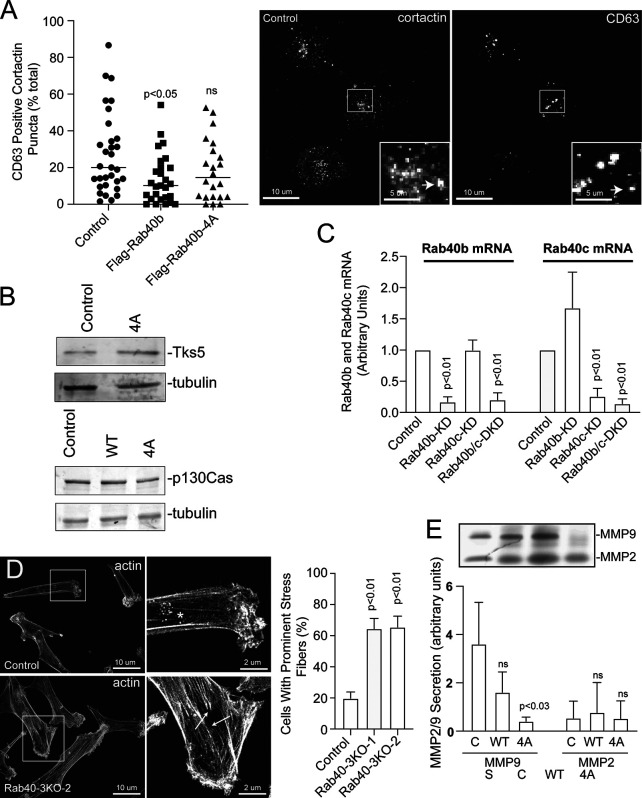
**Stress fiber formation and MMP secretion analysis in Rab40b-4A and Rab40-3KO cells. (A)** Control, FLAG-Rab40b, and FLAG-Rab40-4A cell lines were plated on collagen-coated coverslips and fixed and stained with anti-cortactin (image on left) and anti-CD63 antibodies (image on right). Boxes mark the region that is displayed as higher-resolution image. Arrows point to organelles positive for both cortactin and CD63. Quantification on the left are the means and SEM. **(B)** WB images of Tks5 and p130Cas from control and FLAG-Rab40b-4A cells. Numbers represent densitometry analysis from one biological replicate relative to tubulin and standardized to control levels. **(C)** The knockdown efficiency for Rab40b siRNA and Rab40c siRNA as determined by RT-qPCR. **(D)** Control, Rab40-3KO-1, and Rab40-3KO-2 cell lines were plated on collagen-coated coverslips and then fixed and stained using phalloidin–Alexa Fluor 594. Boxes mark the region that is displayed as higher-resolution image. Arrows point to stress fibers. Quantification on the right shows the means and SEM derived from three independent analyses. A total of 150 cells were analyzed for each condition. **(E)** Gelatin zymography analysis of MMP2 and MMP9 secretion from control, FLAG-Rab40b, and FLAG-Rab40b-4A cells grown in serum-free media for 24 h. FBS (serum) contains MMP2/9 and was used as a positive control. The data shown are the means and SEM derived from three independent experiments. The data were normalized against serum (S) levels of MMP2 and MMP9. DKD, double knockdown.

We next set out to identify the substrates that may be targeted by this complex. Since SOCS box–containing scaffolds recruit proteins for ubiquitylation, we first tested whether known Rab40b-bound proteins, such as Tks5 (Jacob et al., 2016), are degraded in a Rab40b–Cullin5-dependent fashion. To determine that, we analyzed whether overall cellular levels of Tks5 are increased in cells overexpressing FLAG-Rab40b-4A mutants, since that would be expected if Rab40b–Cullin5 mediates Tks5 ubiquitylation and degradation. Surprisingly, the FLAG-Rab40b-4A mutant had little effect on total Tks5 levels compared with control cells (Fig. S3 B), suggesting that Tks5 may not be a substrate for Rab40b–Cullin5 complex ubiquitylation. We next tested if p130Cas, a known FA protein (Cary et al., 1998; Honda et al., 1999; Machiyama et al., 2014) that has been shown to be regulated by Cullin5 (Teckchandani et al., 2014), was affected by loss of Rab40b–Cullin5 binding. As shown in Fig. S3 B, p130Cas is also not stabilized with loss of Rab40b–Cullin5 complex formation, suggesting that the Rab40b–Cullin5 complex may act on an as-of-yet unknown set of proteins.

**Figure 5.**
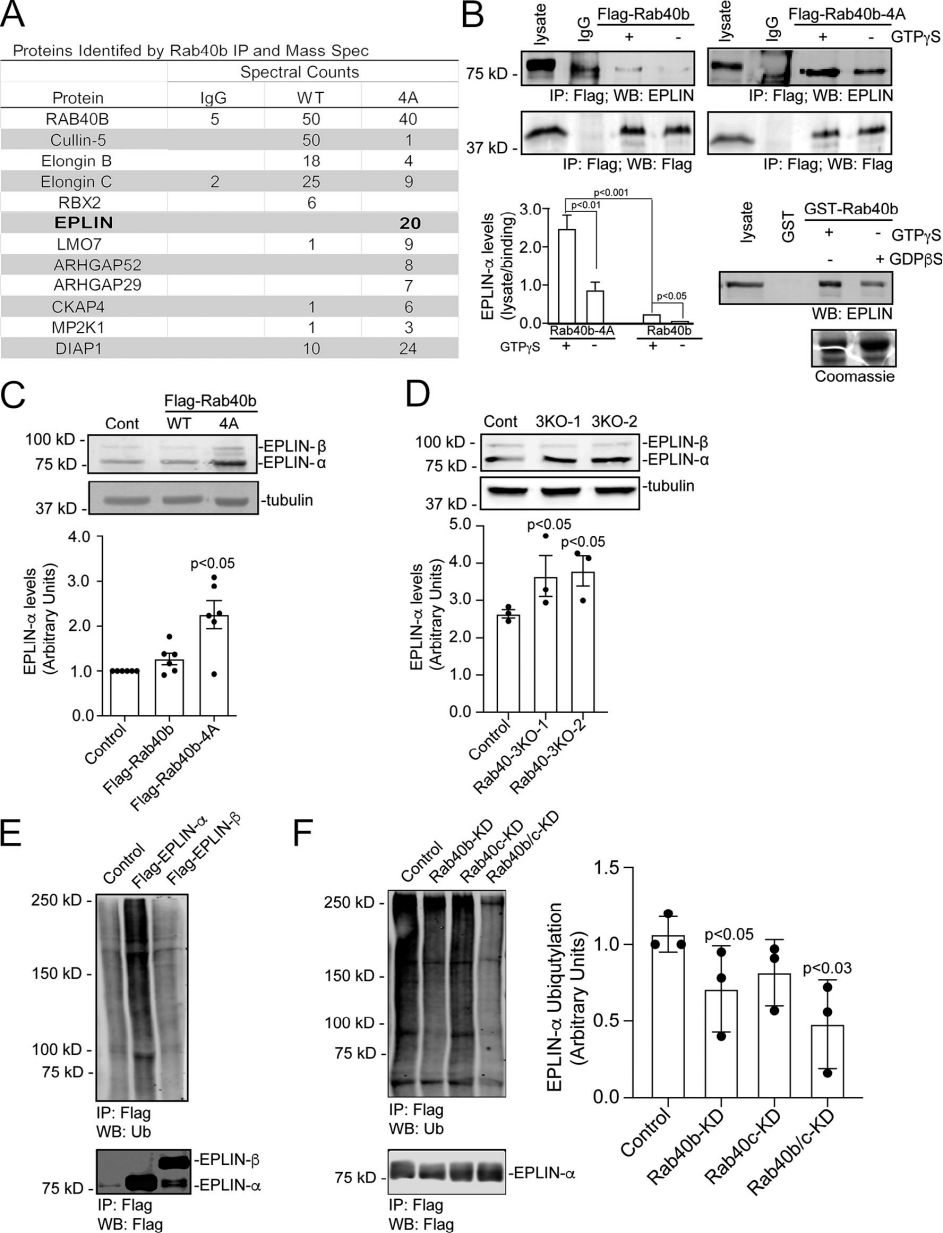
**Rab40b–Cullin5 regulates EPLIN stability and localization.** **(A)** Abbreviated list of proteins identified by IP of FLAG-Rab40b and FLAG-Rab40b-4A cells, followed by mass spectroscopy analysis. **(B)** Rab40b and EPLIN interaction analysis. Top panels: Cell lysates of FLAG-Rab40b and FLAG-Rab40b-4A cells in the presence or absence of GTPγS were incubated with either IgG or an anti-FLAG antibody and immunoprecipitated with Protein G beads. Immunoprecipitates were analyzed by WB with anti-FLAG and anti-EPLIN antibodies. Bottom left: Quantification of EPLIN and FLAG-Rab40b binding. Data are the means and SEM derived from three independent experiments. In all cases, signal was normalized to the EPLIN signal in lysate. Bottom right: MDA-MB-231 control cell lysates were incubated with glutathione beads coated with either GST or GST-Rab40b, in the presence of GDPβS or GTPγS. Bound EPLIN was eluted and analyzed by WB. **(C)** WBs of endogenous EPLIN in control, FLAG-Rab40b, and FLAG-Rab40b-4A cells. Quantification below are the means and SEM derived from three different experiments and normalized against tubulin levels. **(D)** WBs of endogenous EPLIN in control and Rab40-3KO cells. Two different 3KO lines were used in these experiments. Quantification below are the means and SEM derived from three different experiments and normalized against tubulin levels. **(E)** WB images of 293t cells transfected with empty vector control (CNT), FLAG-EPLIN-α, or FLAG-EPLIN-β for 24 h; treated with 10 µm MG132 overnight; harvested and immunoprecipitated for FLAG; and then blotted for either ubiquitin (top) or FLAG (bottom). **(F)** WB images of 293t cells treated with siRNAs for nontargeting control (siCNT), Rab40b, Rab40c, or both Rab40b and Rab40c; transfected with FLAG-EPLIN-α; treated with MG132 overnight; harvested and immunoprecipitated for FLAG; and then blotted for either ubiquitin (top) or FLAG (bottom). Quantification on the right shows the means and SEM derived from three different experiments. KD, knockdown.

Typically, upon ubiquitylation, substrate proteins will rapidly dissociate from the E3 ligase complex, making identification of specific target proteins very difficult. We surmised that certain Rab40b-dependent substrates will remain bound to Rab40b if Cullin5 binding is blocked or diminished, thus allowing us to identify them by mass spectrometry. To that end, we harvested lysates from cells expressing either WT FLAG-Rab40b or mutant FLAG-Rab40b-4A, immunoprecipitated FLAG-Rab40b with anti-FLAG antibodies, and identified bound proteins by mass spectrometry. As shown in Fig. 5 A, Cullin5, Elongin B, and Elongin C coimmunoprecipitation with FLAG-Rab40b-4A was diminished compared with WT FLAG-Rab40b. We also pulled out Rbx2 (known also as Rsf7 or Sag1), a known component of CRL complexes (Fig. 5 A), as a Rab40b–Cullin5-binding protein. To define putative Rab40b–Cullin5 substrates, we next filtered all candidates to focus on proteins that were enriched more than twofold in the FLAG-Rab40b-4A sample compared with WT FLAG-Rab40b (Fig. 5 A).

One highly enriched candidate was EPLIN (also referred to as LIMA1), an actin bundling protein that is known to be a negative regulator of cell migration (Jiang et al., 2008). To test if binding of EPLIN to Rab40b occurs in a GTP-dependent fashion, we again immunoprecipitated FLAG from cell lysates expressing WT or mutant FLAG-Rab40b in the presence or absence of nonhydrolyzable GTP analogue GTPγS and then probed for binding to EPLIN by WB. As shown in Fig. 5 B, EPLIN binds poorly to WT FLAG-Rab40b, consistent with our proteomic analysis. Importantly, Rab40b-4A mutation substantially increased EPLIN’s ability to bind to Rab40b. Finally, Rab40b and EPLIN binding is enhanced by GTPγS (Fig. 5 B). To further confirm that EPLIN binds to Rab40b in GTP-dependent fashion, we next incubated control MDA-MB-231 lysates with glutathione beads coated with purified GST-Rab40b preloaded with either GTPγS or GDPβS. WB analysis of eluted proteins again shows that EPLIN binds with greater affinity to GTP-Rab40b (Fig. 5 B). These data demonstrate that the nucleotide state of Rab40b regulates the binding of EPLIN and thus support the conclusion that EPLIN is a canonical Rab40b effector.

While our data show that EPLIN binds to Rab40b in a manner consistent with classical Rab effectors (Grosshans et al., 2006), its enhanced binding to a Rab40b-4A mutant also suggests that EPLIN might be subject to Rab40b–CRL5-dependent ubiquitylation and that this ubiquitylation may diminish EPLIN’s ability to interact with Rab40b. To test this hypothesis, we first transfected 293t cells with either FLAG-tagged EPLIN-α or EPLIN-β, immunoprecipitated with anti-FLAG antibodies and Western blotted for ubiquitin. As shown in Fig. 5 E, EPLIN-α was ubiquitylated, suggesting that Rab40b–Cullin5 may specifically regulate ubiquitylation of EPLIN-α in these conditions. While we did not detect ubiquitylation of FLAG-EPLIN-β, we cannot discount the possibility that the sensitivity of our assays was not good enough to detect EPLIN-β ubiquitylation or that 293t cells may not recapitulate the signaling environment needed to ubiquitylate EPLIN-β, especially since 293t cells are not a migratory cell type. To test if Rab40b is targeting FLAG-EPLIN-α for ubiquitylation, 293t cells were transfected with FLAG-EPLIN-α and siRNA for Rab40b. Lysates were then immunoprecipitated with anti-FLAG antibodies and blotted for ubiquitin. Surprisingly, depletion of Rab40b (Fig. S3 C) caused only a slight decrease in ubiquitylated EPLIN-α (Fig. 5 F). It has been shown that closely related Rab family members can bind the same effector proteins and have overlapping functions (Junutula et al., 2004; Grosshans et al., 2006; Pavlos and Jahn, 2011; Zhen and Stenmark, 2015). Since most mammalian cells express Rab40c, including MDA-MB-231 cells, we also used siRNA to knock down Rab40c alone as well as both Rab40b and Rab40c in 293t cells (Fig. S3 C) and similarly assessed EPLIN-α ubiquitylation. While loss of Rab40c had no effect, loss of both Rab40b and Rab40c isoforms together caused a further decrease in the ubiquitylation of EPLIN-α (Fig. 5 F). This supports the conclusion that Rab40b can mediate EPLIN ubiquitylation and that other Rab40 isoforms, such as Rab40c, may also contribute to this process.

If Rab40b-dependent EPLIN ubiquitylation targets EPLIN for degradation, it would be expected that expression of FLAG-Rab40b-4A would lead to stabilization of EPLIN in cells. Consistent with this hypothesis, WB analysis shows total levels of EPLIN are significantly increased in Rab40b-4A mutants (Fig. 5 C) compared with control and WT FLAG-Rab40b-expressing cells. To further test the hypothesis that Rab40 may mediate EPLIN ubiquitylation and degradation, we next generated CRISPR-mediated knockout of three Rab40 family members, Rab40a, Rab40b, and Rab40c (Rab40-3KO). As shown in Fig. 5 D, knockout of all three Rab40 isoforms show a similar increase in EPLIN-α compared with control cells. This suggests that in addition to Rab40b, other members of the Rab40 family may compensate for loss of Rab40b in ubiquitylating EPLIN. Thus, in the rest of the study, we used Ran40-3KO for all functional studies.

### Rab40**–**Cullin5 affects actin cytoskeleton by regulating subcellular EPLIN distribution

**Figure 6.**
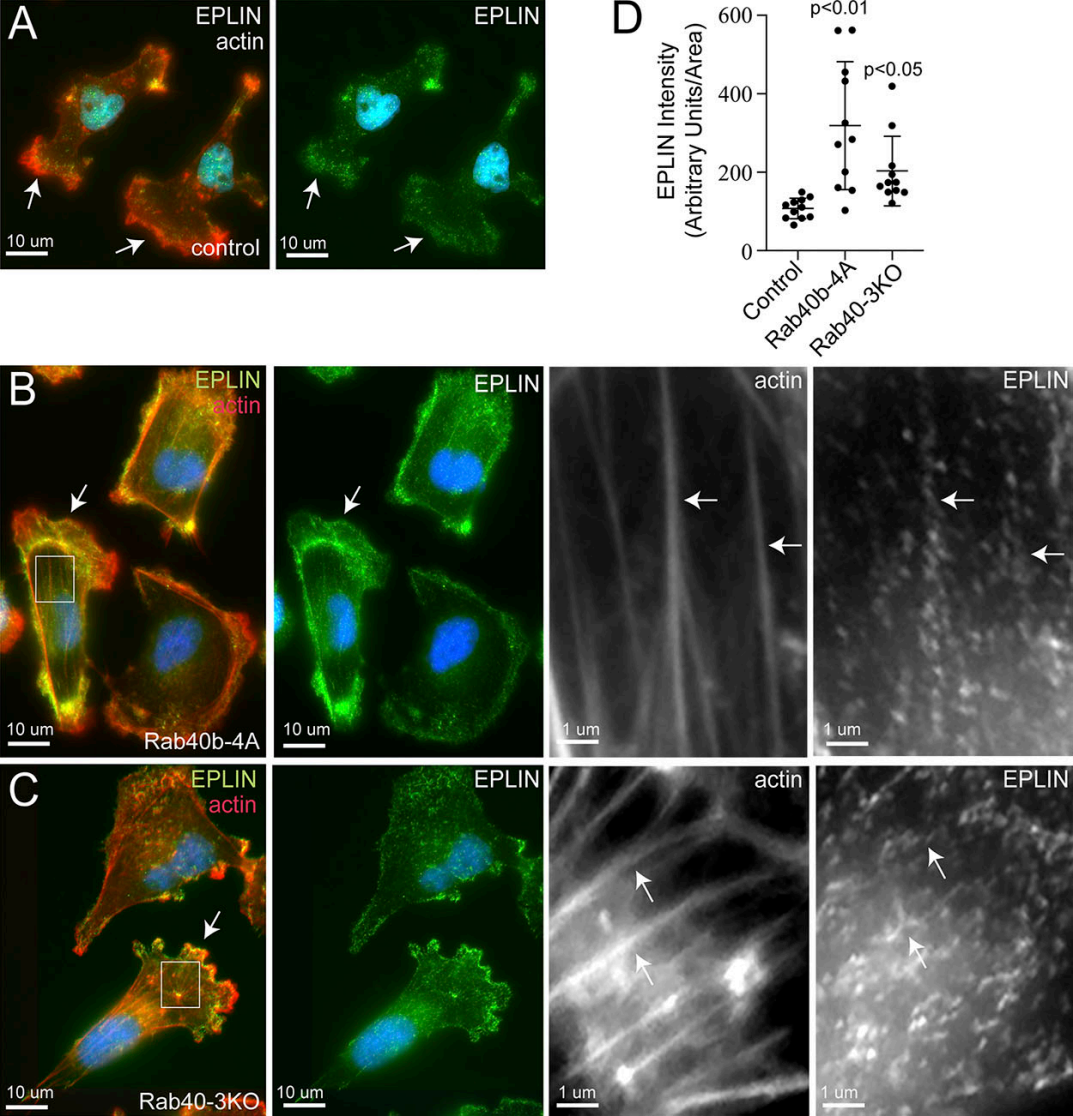
**Rab40a/b/c depletion or Rab40b-4A overexpression leads to increase in plasma membrane– and stress fiber–associated EPLIN. (A–C)** IF images of control (A), FLAG-Rab40b-4A (B), and Rab40-3KO (C) cells plated on collagen-coated coverslips and then fixed and stained with anti-EPLIN antibodies (green) and phalloidin–Alexa Fluor 594 (red). Boxes mark regions of interest. Arrows in whole-field images indicate the lamellipodia leading edge. Arrows in boxed regions indicate stress fibers. **(D)** Quantification of EPLIN fluorescence in control, FLAG-Rab40b-4A, and Rab40-3KO cells. The data shown are the means and SEM derived from three different experiments. Dots represent individual cells analyzed.

**Figure 7.**
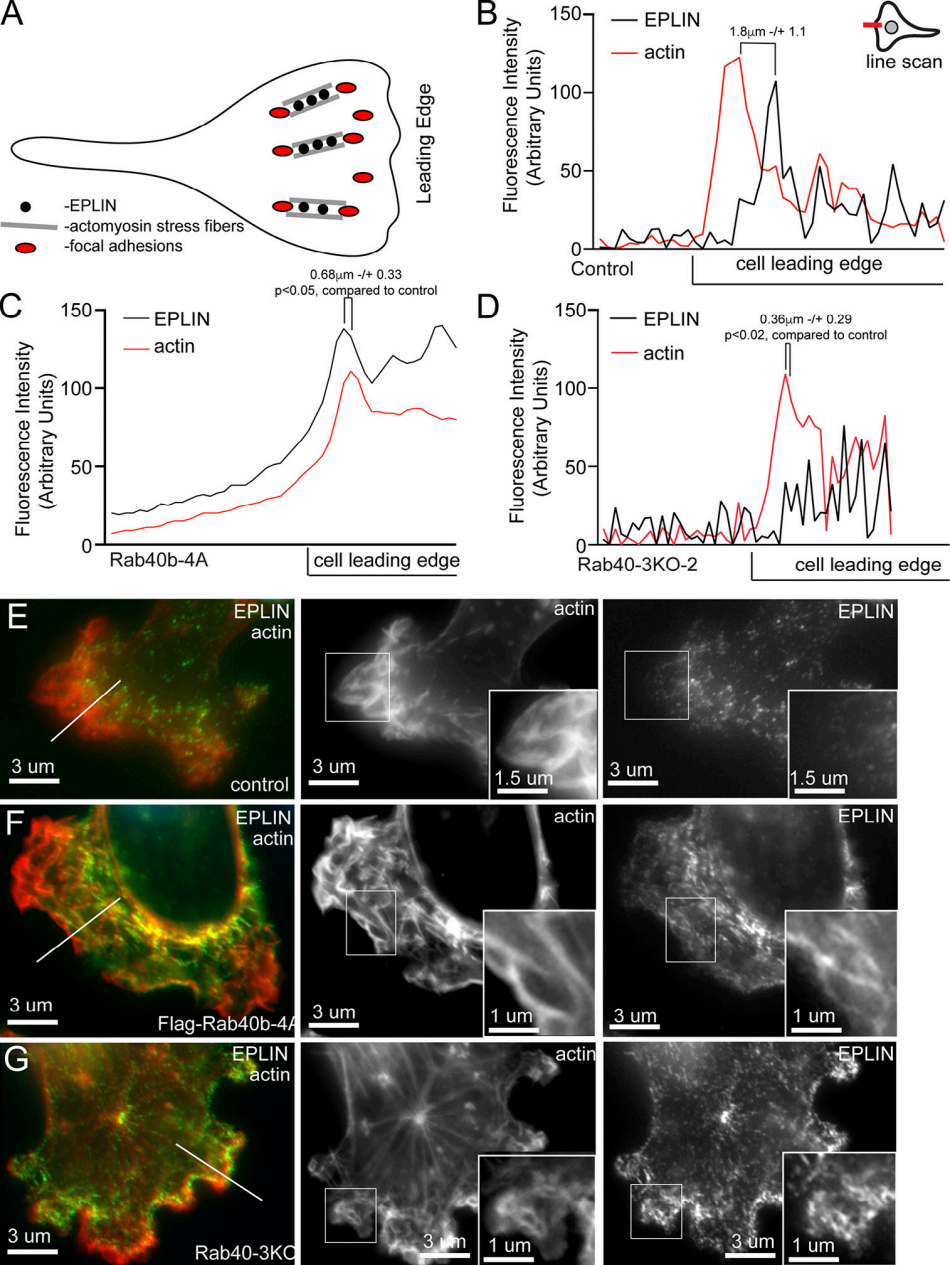
**Rab40b inhibits EPLIN accumulation at the lamellipodia leading edge.** **(A)** Schematic representation of FA and stress fiber distribution in lamellipodia. **(B–G)** Representative line scans from control (E), FLAG-Rab40b-4A (F), and Rab40-3KO (G) cells plated on collagen-coated coverslips and then fixed and stained with phalloidin–Alexa Fluor 594 (red) and anti-EPLIN (green) antibodies. Boxes mark the region of interest shown in inset. Line marks the region analyzed by line scan (quantifications shown in B–D). EPLIN distance from actin front: control, 1.8 µm ± 1.1; FLAG-Rab40b-4A, 0.68 µm ± 0.33 (P < 0.05); and Rab40-3KO, 0.36 µm ± 0.29 (P < 0.02). *n* = 5 cells for each cell line.

EPLIN targeting to specific subcellular domains was shown to play a key role in regulating EPLIN function (Taha et al., 2019). As shown in Fig. 6 A, immunofluorescent (IF) staining with a pan-EPLIN antibody shows that in control MDA-MB-231 cells, EPLIN is localized at the lamellipodia, just behind lamellipodia actin ruffles (also see Fig. 7 E). This is consistent with the proposed function of EPLIN in the regulation of actin bundling during formation of actoMyosin stress fibers that are localized just behind leading edge of lamellipodia and are required for directional cell migration (Fig. 7 A; Shellard and Mayor, 2021). In contrast, in FLAG-Rab40b-4A and Rab40-3KO cells, EPLIN strongly associates with stress fibers (Fig. 6, B and C) and was present at the leading edge of the lamellipodia (Fig. 7, B–G). Interestingly, lamellipodia in FLAG-Rab40b-4A and Rab40-3KO cells were smaller and had less pronounced actin ruffles, an observation consistent with reports that EPLIN-induced actin filament bundling may inhibit Arp2/3-dependent actin ruffling (Taha et al., 2019; Maul et al., 2003).

**Figure 8.**
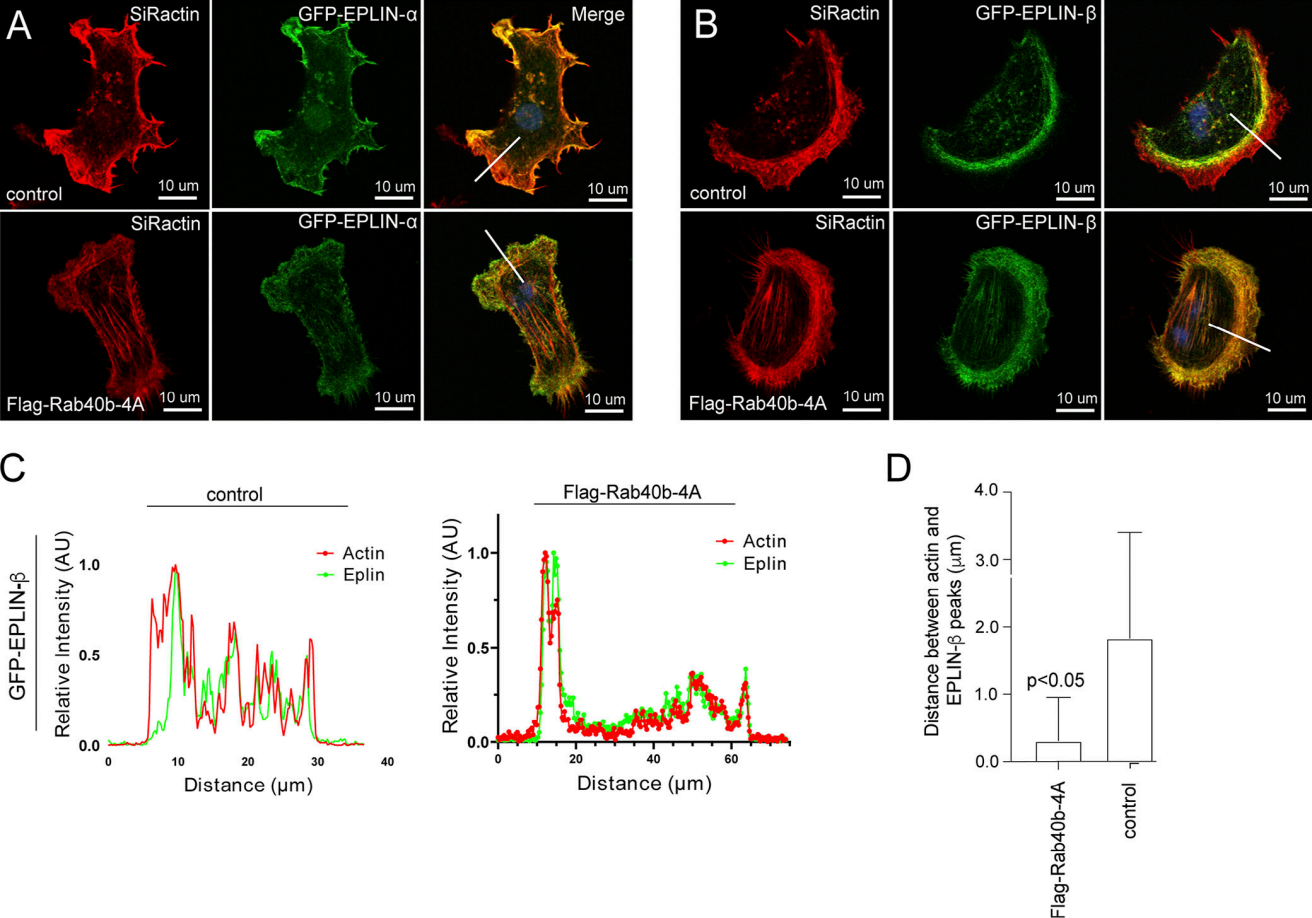
**Rab40b regulates subcellular localization of EPLIN-β.** **(A)** Control or FLAG-Rab40b-4A–expressing cells were transfected with either GFP-EPLIN-α (green) or GFP-EPLIN-β (green). Cells were then fixed and stained with phalloidin–Alexa Fluor 594 (red). Line indicates the area analyzed by line scan and shown in B. **(B and C)** EPLIN and actin distribution in lamellipodia was analyzed by line scan. The location of line-scan analysis is shown in A. Panel C shows the analysis of the distance between actin front at the leading edge and EPLIN front. The data shown are the means and SEM derived from five different cells. **(D)** Representative images of immunohistological stains of EPLIN from tumors grown from each cell line. AU, arbitrary units.

Because recent work has suggested differences in localization and function for each EPLIN isoform (Jacquemet et al., 2019; Taha et al., 2019), we next asked if the subcellular localization changes of EPLIN we observed were due to differential regulation of individual isoforms. We transfected control or FLAG-Rab40b-4A cells with GFP-EPLIN-α or GFP-EPLIN-β. Although stress fibers are generally absent in control MDA-MB-231 cells, both isoforms are present along the stress fibers induced by FLAG-Rab40b-4A mutant cells (Taha et al., 2019). Additionally, in control cells, GFP-EPLIN-β localizes just behind the actin-rich front edge of lamellipodia (Fig. 8, A and B). Similar to endogenous GFP-EPLIN distribution, in Rab40b-4A cells, GFP-EPLIN-β no longer lags behind the actin ruffles in lamellipodia, which suggest that Rab40b–Cullin5 binding influences localization of EPLIN-β during cell migration (Fig. 8, A–D).

### Rab40**–**Cullin5 regulates lamellipodia dynamics during cell migration

**Figure 9.**
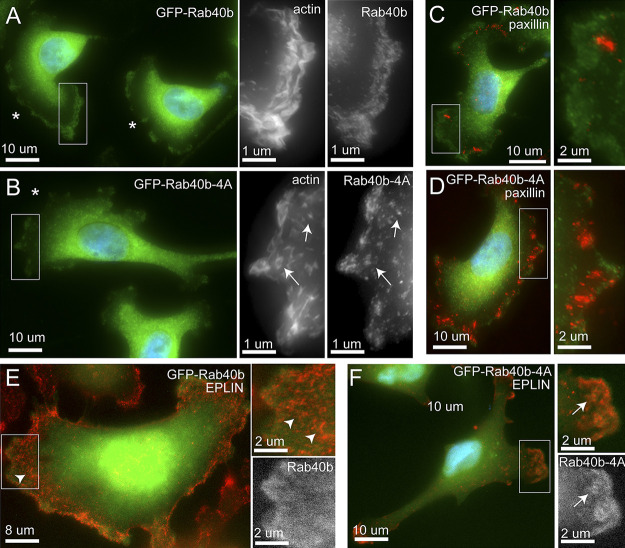
**GFP-Rab40b colocalizes at the actin ruffles at the leading edge of lamellipodia.** **(A–F)** Representative images of GFP-Rab40b (A, C, and E) or GFP-Rab40b-4A (B, D, and F) expressing MDA-MB-231 cells plated on collagen-coated coverslips and then fixed and stained with phalloidin–Alexa Fluor 594 (see insets in A and B), anti-paxillin (C and D), or anti-EPLIN antibodies (E and F). Boxes mark the region of interest shown in the inset. Asterisks mark the leading edge of lamellipodia. Arrowheads mark EPLIN staining that does not colocalize with GFP-Rab40b. Arrows point to structures positive for both EPLIN and GFP-Rab40b-4A.

We next sought to analyze the subcellular distribution of Rab40b to better understand where the Rab40b–Cullin5 complex may function to regulate EPLIN ubiquitylation and degradation. To that end, we generated MDA-MB-231 cell lines stably expressing either GFP-Rab40b or GFP-Rab40b-4A. As shown in Fig. 9 A, while the majority of GFP-Rab40b is present in the cytosol, a subpopulation of GFP-Rab40b can clearly be observed at the lamellipodia, where it colocalizes with actin ruffles. GFP-Rab40b-4A is also present at the front end of lamellipodia and colocalizes with actin, suggesting that inhibition of Cullin5 binding does not affect subcellular localization of Rab40b (Fig. 9 B). As was the case with our aforementioned data, GFP-Rab40b-4A–expressing cells had diminished levels of actin ruffles (Fig. 9 B) and an increase in FAs compared with the cells expressing WT GFP-Rab40b (Fig. 9, C and D). Similarly, in GFP-Rab40b-4A cells, EPLIN accumulates at the leading edge of lamellipodia, where it colocalizes with GFP-Rab40b-4A, an association largely absent in cells expressing WT GFP-Rab40b (Fig. 9, E and F).

**Figure 10.**
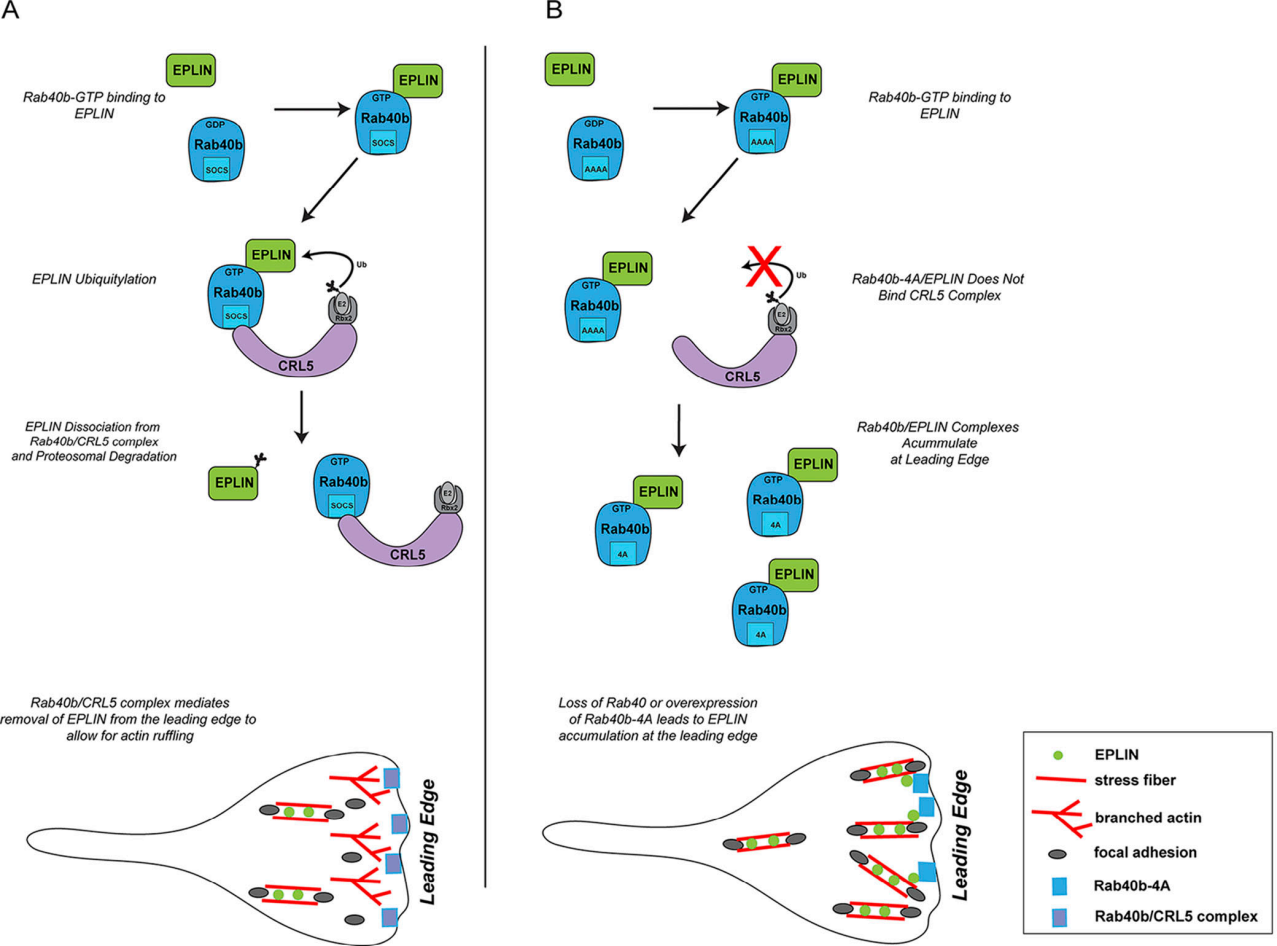
**Proposed model for Rab40b function during cell migration.** **(A)** GTP-bound Rab40b binds to EPLIN. Rab40b/EPLIN is then recognized by the CRL5 complex via the Rab40b SOCS box. Binding of EPLIN with the Rab40b–CRL5 complex leads to EPLIN ubiquitylation, disassociation of EPLIN of Rab40b and EPLIN complex, and eventual degradation by the proteasome. Rab40b is enriched at the leading edge of lamellipodia, which leads to the exclusion of EPLIN from lamellipodia, thus allowing actin ruffling. **(B)** Rab40b-4A mutation blocks Rab40b association with the CRL5 complex, leading to inhibition of EPLIN ubiquitylation and stabilization of the Rab40b-4A–EPLIN complex. Consequently, EPLIN accumulates at the leading edge of the lamellipodia, thus resulting in inhibition of actin ruffling and an increase in actoMyosin stress fibers.

**Figure S4.**
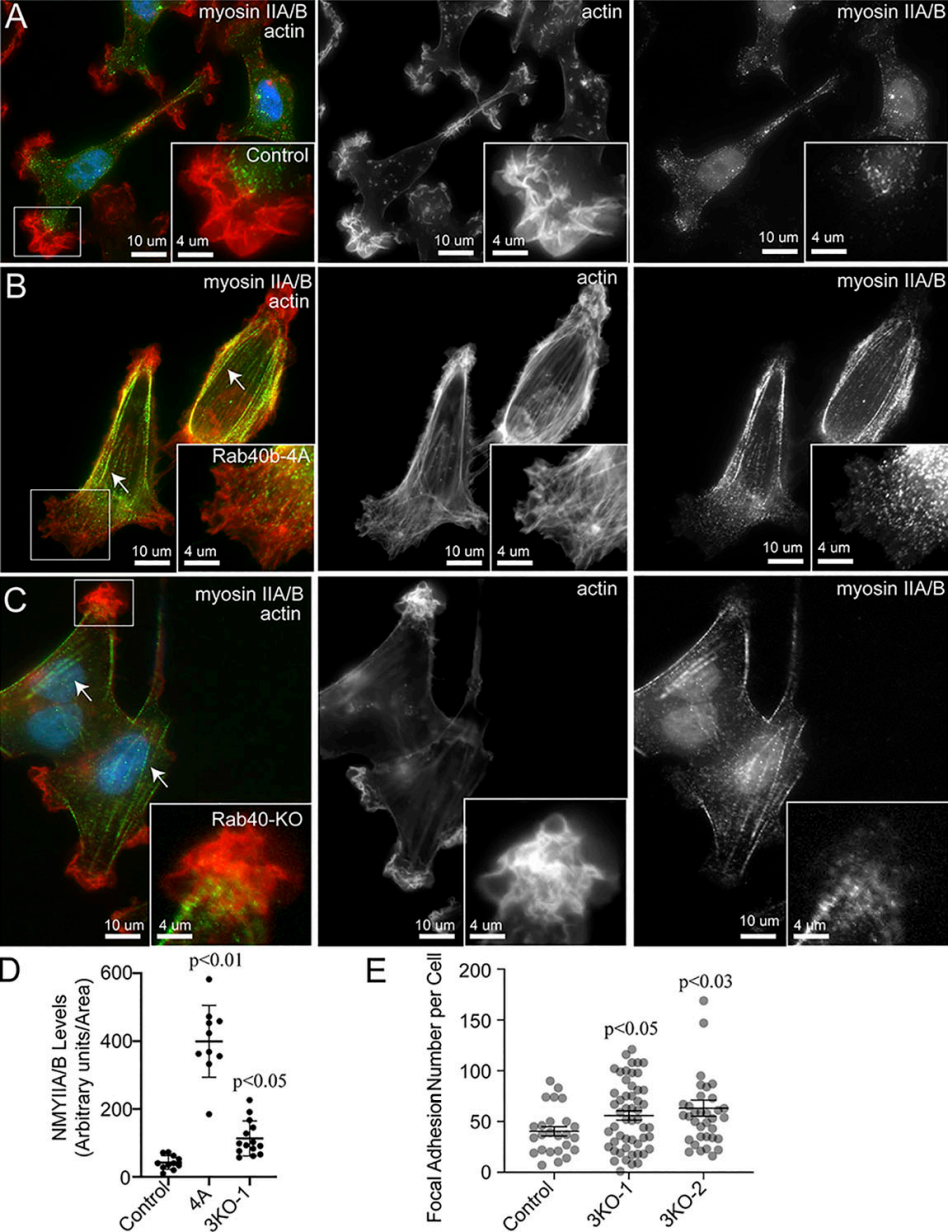
**The effect of Rab40 in regulating non-muscle Myosin IIA/B. (A–D)**Control, Rab40b-4A, and Rab40-3KO cell lines were plated on collagen-coated coverslips, fixed and stained using phalloidin–Alexa Fluor 594 and anti-nonmuscle Myosin IIA/B antibodies. Boxes mark the region that is displayed as higher-resolution image. Arrows point to stress fibers. Quantification in D shows the means and SEM derived from three independent analyses. Dots represent individual cells analyzed. **(E)** Quantification of number of FAs per cell for control, Rab40b-3KO-1, and Rab40b-3KO-2 cells. *n* ≥ 20 cells per condition. NMY, nonmuscle Myosin.

**Figure S5.**
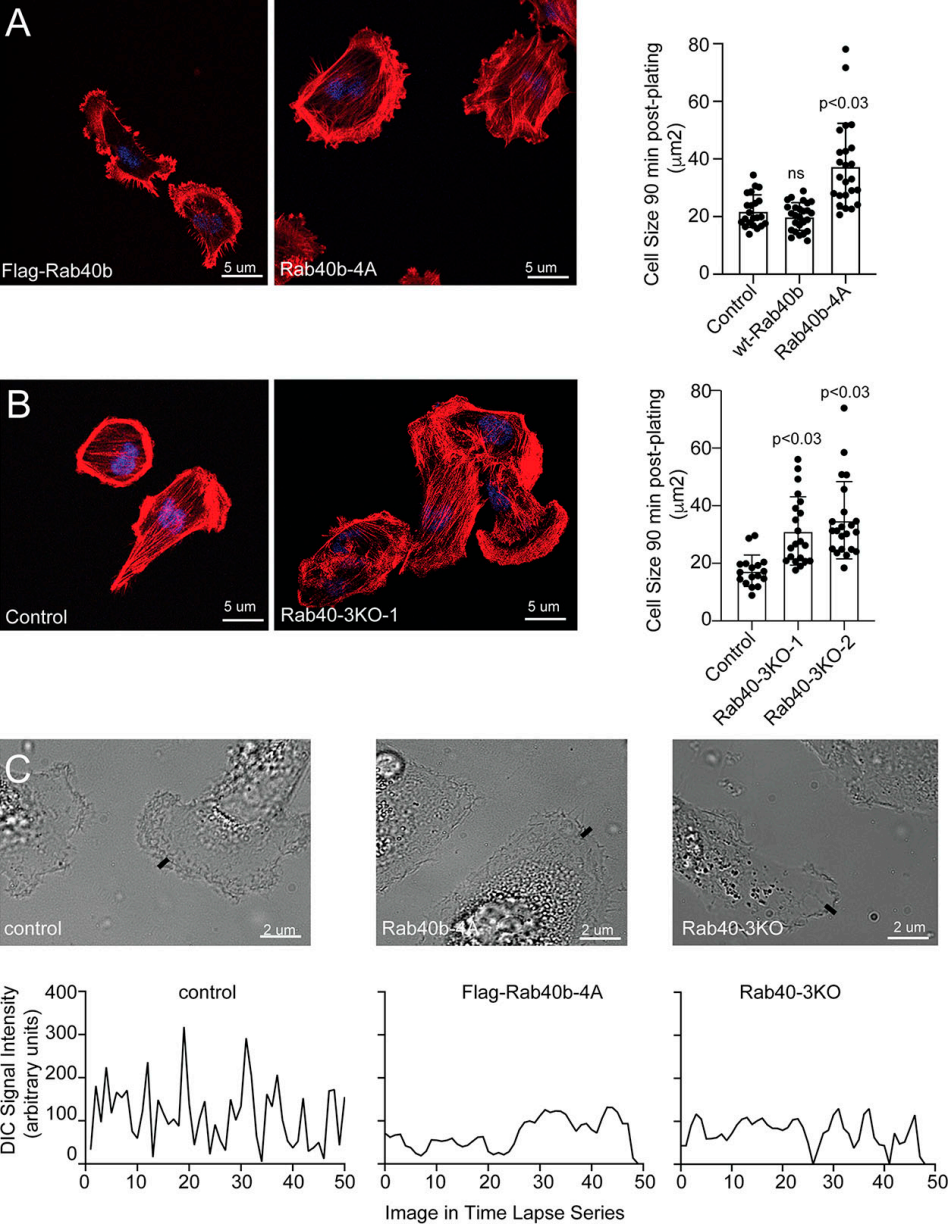
**The role of Rab40 in regulating cell–ECM adherance and lamellipdia dynamics. (A and B)**Control, FLAG-Rab40b, Rab40b-4A, and Rab40-3KO cell lines were seeded on collagen-coated coverslips and incubated for 90 min. Cells were then fixed and stained using phalloidin–Alexa Fluor 594. Quantification on the right shows the means and SEM derived from three independent analyses. Dots represent individual cells analyzed. **(C)** Control, FLAG-Rab40b-4A, and Rab40-3KO cell lines were seeded on collagen-coated coverslips. Invadopodia dynamics was then imaged by time-lapse microscopy/DIC. Small line marks the location of quantification shown on the bottom. To visualize the dynamics of the entire lamellipodia, see Videos 5, 6, and 7.

EPLIN has a well-established role in inhibiting Arp2/3 branched actin polymerization (Maul et al., 2003; Taha et al., 2019), a process known to be essential for actin ruffling at the leading edge of cells. The accumulation of EPLIN and altered actin ruffles in our Rab40b mutant cells suggests altered lamellipodia dynamics. To that end, we imaged lamellipodia plasma membrane dynamics using differential interference contrast (DIC) time-lapse microscopy. As expected, control cells exhibited very active lamellipodia ruffling (Fig. S5 C and Video 5). In contrast, overexpression of FLAG-Rab40b-4A or depletion of Rab40a/b/c (Rab40-3KO) led to decrease in lamellipodia ruffling (Fig. S5 C and Videos 6 and 7). Together, these data suggest that Rab40b mediates removal of EPLIN at the leading edge of migrating cells, whereas expression of mutant Rab40b-4A inhibits EPLIN ubiquitylation, thus stabilizing Rab40b and EPLIN interaction and leading to the accumulation of Rab40b–EPLIN complexes at the leading edge of lamellipodia and stress fibers (see the proposed model in Fig. 10). Importantly, Rab40-3KO phenocopies the effects of overexpressing FLAG-Rab40b-4A, where we observe an increased number of FAs (Fig. S4 E), stimulation of stress fibers (Fig. S3 D), and increased cell–ECM adhesion (Fig. S5 B). If depletion of Rab40a/b/c or overexpression of Rab40b-4A stabilizes stress fibers, one would predict that that should also lead to an increase in stress fiber–associated nonmuscle Myosin IIA/B. We therefore stained MDA-MB-231 cells with anti-nonmuscle Myosin IIA/B antibodies. Consistent with our hypothesis, FLAG-Rab40b-4A and Rab40-3KO cells exhibited increase in stress fiber–associated nonmuscle Myosin IIA/B (Fig. S4, A–D).

**Video 5.** **MDA-MB-231 cells were plated on collagen-coated glass-bottom dishes and imaged by DIC.** 50 images with time lapse of 3 s were taken for each cell. Playback rate, 0.25 frames per second.

**Video 6.** **FLAG-Rab40b-4A expressing cells were plated on collagen-coated glass-bottom dishes and imaged by DIC.** 50 images with time lapse of 3 s were taken for each cell. Playback rate, 0.25 frames per second.

**Video 7.** **Rab40-3KO cells were plated on collagen-coated glass-bottom dishes and imaged by DIC.** 50 images with time lapse of 3 s were taken for each cell. Playback rate, 0.25 frames per second.

Overall, our results suggest that Rab40b complexes with Cullin5 to regulate subcellular EPLIN localization by decreasing EPLIN levels at the leading edge of lamellipodia and allowing EPLIN accumulation at stress fibers and invadopodia. It is likely, however, that EPLIN is only one of several Rab40b substrates and that Rab40b–Cullin5 regulates the activity of multiple actin regulators. Accordingly, our proteomic screen identified several other regulators of actin dynamics that are putative substrates for the Rab40b–Cullin5 complex (Fig. 5 A).

## Discussion

The coupled acts of cell migration and invasion involve very complex and highly interconnected molecular pathways that must be properly coordinated to ensure correct organism development and function. Previous reports from our laboratory have identified Rab40b as an important protein for the secretion of MMP2/9 at invadopodia and central for cell invasion and migration (Jacob et al., 2013; Jacob et al., 2016; Jacob and Prekeris, 2015). Rab40b belongs to a unique Rab40 subfamily of small monomeric GTPases that contain SOCS domain at their C terminus, just before the geranylgeranylation site. SOCS domains are known to mediate interaction with CRLs; thus, we hypothesized that Rab40b may mediate its function by regulating protein ubiquitylation. While it has been shown that Rab40a and Rab40c bind Cullin5, it remains unclear whether the same is true for Rab40b. Here, we sought to further understand how Rab40b impacts other aspects of cell invasion by exploring two open questions: does Rab40b–Cullin5 binding play a role in regulating cell migration and invasion, and what are the substrates of this complex?

We show that in breast cancer cells Rab40b does bind to the CRL5 E3 ligase complex containing Cullin5, Elongin B, Elongin C, and Rbx2 and that mutations to the Rab40b–SOCS box are sufficient to disrupt this interaction. We also demonstrate that loss of the Rab40b–Cullin5 complex by Rab40b-4A mutation impacts chemotactic migration and invasion. Analysis of individual cell movement also revealed defects and alterations in cell behavior. While cell speed remains unchanged, there are noticeable differences in cell directionality, which suggests that the deficiency of Rab40b-4A–expressing cells to move toward a chemoattractant may be due to a loss in directionality. Mutant cells also have less invadopodia and have a decreased ability to move through a 3D ECM environment. Interestingly, our time-lapse analyses also suggest that Rab40b-4A cells have increased cell–cell and cell–ECM adherence, thus leading to formation of cell clumps and apparent loss of contact inhibition of locomotion.

Mutant Rab40b-4A and Rab40-3KO cells also adhere more strongly to the ECM and exhibit an increase in ventral stress fibers. Previous reports have shown that stress fibers can be induced in MDA-MB-231 cells by activating RhoA (Maeda et al., 2011; Gilkes et al., 2014), suggesting that Rab40b-4A cells may have altered RhoA signaling. We also observe an increase in both the number and size of FAs per cell, with an accompanying increase in total paxillin, as well as more zyxin-positive FAs. This suggests that FAs are more stable in mutant Rab40b-4A and Rab40-3KO cells. Indeed, live-imaging analysis shows that adhesions have a greater life span in Rab40b-4A cells. It was recently shown that Rab18 regulates FA dynamics in U2OS cells. Rab18 is closely related to the Rab40 family (Pereira-Leal and Seabra, 2001), and it has been suggested that Rab40 split from the Rab18 family and gained the SOCS box upon the development of multicellularity, so it is not surprising that such closely related isoforms would regulate similar cellular processes. Localization of FAs is tightly controlled by migrating cells, with coordinated assembly and turnover of FAs at the front of the cell body and release of adhesions at the rear. In Rab40b-4A and Rab40-3KO cells, FAs are present throughout the cell and do not appear to be enriched specifically at leading lamellipodia, which helps explain difficulties in maintaining cell directionality.

Signaling through FAK is complex, but recent reports have demonstrated its importance for regulating cell invasion. We observe differences in pFAK signaling in FLAG-Rab40b-4A cells, which have greater levels of Y397 and less S910 compared with control and FLAG-Rab40b cells. This result is in line with observations that phosphorylation of FAK at Y397 stabilizes FAs and decreases both invadopodia and ECM degradation and that phosphorylation at S910 is associated with FA turnover, ECM degradation, and metastasis. While initially required to relay extracellular signaling cues, FAs must disassemble not only to generate the force needed for cell migration but also to free up signaling components required for invadopodia function (Kolli-Bouhafs et al., 2014; Badowski et al., 2008). Taken together, our data demonstrate that the Rab40b–Cullin5 complex regulates cell migration and invadopodia formation, in part by modulation of FA dynamics and FAK signaling, and are consistent with a model wherein Rab40b is central to regulating various aspects of cell migration and invasion.

The main function of SOCS box–containing proteins is to serve as an adaptor to Cullin5 to mediate ubiquitylation of specific substrate proteins. Identification of these substrates is a key step to understand how Rab40b–Cullin5 mediates cell migration and invasion. WB analysis of known CRL5 substrates showed no differences in stabilization in Rab40b-4A cells and suggests that the Rab40b–Cullin5 complex acts to regulate a unique set of proteins. In an effort to identify possible substrates, we completed comparative proteomic analysis of proteins that bind to either to WT FLAG-Rab40b or FLAG-Rab40b-4A. We speculated that a subset of Rab40b-bound substrates would not get ubiquitylated in the absence of CRL5 binding and thus remain bound to Rab40b. Consistent with this idea, we identified several proteins that were enriched in mutant FLAG-Rab40b-4A immunoprecipitates compared with WT. In this study, we focused on EPLIN, an actin bundling protein and a well-established tumor suppressor (Wu, 2017). Interestingly, EPLIN also binds to Rab40b in a GTP-dependent fashion. This raises an intriguing possibility that EPLIN acts as a canonical Rab40b effector protein (Fig. 10).

While it has been reported that EPLIN turnover occurs in response to growth factor stimulation (Zhang et al., 2013), the molecular machinery that facilitates this process is not known. Here, we show that Rab40b binds to Cullin5 and that loss of Rab40b decreases levels of ubiquitin-conjugated EPLIN, thus leading to a decrease in EPLIN degradation. This suggests a model in which EPLIN binds Rab40b, which in turn binds Cullin5 and the remaining components of the CRL5, leading to ubiquitylation of EPLIN (Fig. 10 A). The EPLIN ubiquitylation then leads to dissociation of Rab40b–EPLIN complex and subsequent EPLIN degradation (Fig. 10 A). Although upstream signaling mechanisms that trigger EPLIN ubiquitylation have been reported, to our knowledge, we are the first to identify specific molecular components responsible for EPLIN ubiquitylation and degradation. In addition to proteasomal degradation, cells use ubiquitylation of target proteins to regulate activity and localization, and we observe that Rab40b–Cullin5 alters the localization of EPLIN to the leading edge of lamellipodia, presumably by localized EPLIN ubiquitylation. Indeed, the addition of ubiquitin as a standard posttranslational modification has been shown to regulate localization and activity of some target proteins (Shin et al., 2017; Ameka et al., 2014). Interestingly, we could only detect Rab40-dependent ubiquitylation of EPLIN-α isoform. Although we have not directly addressed the mechanism that determines the discrepancy in regulatory fates of EPLIN isoforms, it is possible that our assays were simply not sensitive enough to detect transient ubiquitylation in EPLIN-β. Alternatively, since it has been suggested that EPLIN-α expression regulates EPLIN-β, it could be that EPLIN-α ubiquitylation also indirectly affects EPLIN-β function and localization.

The high sequence similarity among the Rab40 family members makes it likely that other Rab40 isoforms may play a role in EPLIN binding, stabilization, or localization. Indeed, the fact that Rab40b/Rab40c double knockdown further decreased EPLIN-α ubiquitylation (as compared with Rab40b knockdown) suggests that the entire Rab40 family may be involved in regulating EPLIN. Consistent with this hypothesis, only cells lacking Rab40a, Rab40b, and Rab40c isoforms phenocopy defects observed in cells expressing FLAG-Rab40b-4A. The FLAG-Rab40b-4A mutant stabilizes the Rab40b and EPLIN interaction, and its expression likely leads to formation of Rab40b–EPLIN complexes that are unrecognized by CRL5 components. The accumulation of such Rab40b complexes with EPLIN or other substrates makes it likely that mutant Rab40b-4A acts in a dominant-negative fashion with respect to Cullin5 binding. Ascertaining substrate specificity and overlap for different Rab40 isoforms, as well as how Rab40/substrate interactions impact cellular function, will be the focus of our future work.

Here, we show that Rab40b localizes to lamellipodia, where it colocalizes with actin ruffles, independent of Cullin5-binding capability, and that these actin ruffles are diminished upon expression Rab40b-4A or depletion of Rab40a/b/c. Coincident with a decrease in lamellipodia actin ruffles, we observe an accumulation of EPLIN at the leading edge and a decrease in lamellipodia dynamics. The ability of EPLIN to bundle actin filaments and decrease actin dynamics, together with its regulation by Rab40b–Cullin5, suggests to us that it is likely Rab40b–Cullin5-dependent ubiquitylation is needed to remove and exclude EPLIN from the leading edge for efficient cell migration to occur. Consistent with this hypothesis, overexpression of Rab40b-4A or knockout of Rab40a/b/c leads to accumulation of EPLIN at the leading edge, thus leading to a decrease in leading edge dynamics. We propose that rather than regulating global cellular EPLIN levels, Rab40b regulates EPLIN function via localized ubiquitylation-dependent removal and degradation from the leading edge of the cell (Fig. 10).

In contrast to previous reporting (Maul et al., 2003), overexpression of either EPLIN isoform was not sufficient to drive cells to a prominent stress fiber phenotype in our system. In addition to inherent cell type–specific differences, this suggests that other molecular factors in addition to EPLIN contribute to the observed phenotypes in Rab40b-4A mutant or Rab40-3KO cells. Interestingly, the majority of proteins that exhibit increased binding to Rab40b-4A are regulators of actin cytoskeleton dynamics, suggesting that the Rab40b–Cullin5 complex targets a specific subset of actin regulators to effect actin dynamics during cell migration (Fig. 5 A). For example, ARHGAP42 (Luo et al., 2017) and ARHGAP19 (David, Petit, and Bertoglio 2014) both regulate RhoA activation. LMO7 is a LIM domain–containing protein implicated in the regulation of actin dynamics at FAs and adherens junctions (Ooshio et al., 2004). Finally, DIAPH1 is an actin nucleating factor that regulates actin polymerization in response to RhoA activation (Li and Sewer 2010). While further studies will be needed to confirm and identify specific roles of these Rab40b-binding proteins, the common feature among them all is that they are involved in the regulation of RhoA signaling and actin dynamics.

Taken together, our data suggest that Rab40b is a unique small monomeric GTPases that appears to have two distinct functions. On one hand, Rab40b may function as a canonical Rab GTPase by regulating MMP2/9-containing secretory vesicle targeting to invadopodia (Bravo-Cordero et al., 2007; Wiesner et al., 2013; Hoshino et al., 2013). On the other hand, Rab40b acts as a SOCS-adaptor protein for CRL5 and mediates Cullin5-dependent protein degradation. These two functions seem to converge on the same downstream goal to regulate subcellular localization of specific actin regulators and promote directional cell migration and invasion. Many questions, however, still remain. How is Rab40b and EPLIN binding regulated? Does Rab40b regulate cell migration during development in vivo? How is Rab40b targeted to the leading edge of lamellipodia? What are the functions of other Rab40 isoforms and substrates? Does the Rab40b–CRL5 complex also directly regulate MMP secretion? Finally, all of the experiments in this study were done using a single breast cancer cell line, MDA-MB-231. Thus, it remains to be seen whether Rab40 GTPases have similar effects on actin and FA dynamics in different breast cancer cell lines or cells derived from other cancers. Additional work will be needed to address these questions and will be the focus of future studies.

## Materials and methods

### Cell culture and cell lines

All cell lines were cultured as described previously (Jacob et al., 2013). The MDA-MB-231 cell line stably expressing FLAG–Rab40b-4A was created by cloning Rab40b-4A (primers purchased from Integrated DNA Technologies) into lentiviral pCS2-FLAG vector obtained from Addgene. Cell lines were routinely tested for mycoplasma. All cell lines used in this study were authenticated and are in accordance with American Type Culture Collection standards. For all FLAG-Rab40b and FLAG-Rab40b-4A experiments, we used parental MDA-MB-231 cells as controls. For all Rab40-3KO experiments, MDA-MB-231 cells expressing tetracycline-inducible Cas9 were used as a control.

### Plasmids

GFP-Paxillin, GFP-EPLIN-α, and GFP-EPLIN-β were purchased from Addgene (50529, 40948, and 40947). For FLAG-Rab40b-4A, mutants were generated from an existing PLVX-FLAG-Rab40b plasmid (Jacob et al., 2013). Point mutations for 213L and 215T were created by site-directed mutagenesis with the following primers: 213L forward (5′-GTG​GAC​AAG​CTC​CTG​CTC​CCC​ATT​GCC-3′), 213L reverse (5′-GGG​AAT​GGG​GAG​CAG​CTT​GTC​CAC-3′), 215T forward (5′-GAC​AAG​CTC​CCG​CTC​ACC​ATT​GCC​TAA-3′), and 215T reverse (5′-CTT​AAG​GCA​ATG​GTG​AGC​GGG​AGC​TTG-3′).

For the FLAG-Rab40b-4A mutation, the entire coding sequence for Rab40b containing the AAAA mutation (CTCCCGCTCCCC → GCAGCAGCA) was ordered from Integrated DNA Technologies on a Pbluescript plasmid and subcloned into an existing PLVX-FLAG-Rab40b plasmid. Lentiviral transfection of Rab40b mutant plasmids into MDA-MB-231 cells was then performed as previously described (Jacob et al., 2013). Briefly, cells were transfected using Lipofectamine 2000, following manufacturer protocol. Cells were allowed to recover in serum-supplemented media for 24 h and then used for the experiments.

### Antibodies

The following antibodies were used in this study: anti-FLAG (WB 1:1,000, F3165; Sigma), anti-EPLIN (WB 1:1,000, IF 1:100, 50311; Cell Signaling Technology), anti-EPLIN (WB 1:1,000, IF 1:100, sc-136399; Santa Cruz Biotechnology), anti-EPLIN (WB 1:1,000, immunoprecipitation [IP] 1 μg/1 mg lysate, immunohistochemistry [IHC] 1:200, 16639–1-AP; Proteintech), anti-HA (WB 1:500, IP 2 µg/1 mg cell lysate, SC F-7; Santa Cruz Biotechnology), Alexa Fluor 568–phalloidin (IF 1:1,000, A1238; Life Technologies), SiR-actin (1:10,000, CY-SC001; Cytoskeleton), anti–β-tubulin (WB 1:2,500, 926–42211; LI-COR), anti–α-tubulin (WB 1:2,500, 23948; Santa Cruz Biotechnology), pFAK Y397 (WB 1:1,000, ab8129; Abcam), FAK S910 (WB 1:1,000, 44-596G; Invitrogen), anti–E-Cadherin (WB 1:1,000, IHC 1:200, 3195; Cell Signaling Technology), total FAK (WB 1:1,000, 610087; BD Biosciences), anti-zyxin (IF 1:1,000, ab50391; Abcam), anti-Rab40b (WB 1:500, LS-C353287; LSBio), anti-Rab40c (WB 1:500, H-8 sc-514826; Santa Cruz Biotechnology), and anti-CD63 (IF 1:100; gift from Andrew Peden, University of Shefield, Shefield, UK).

### 3D inverse invasion assay

Assays were adapted and performed as previously described. In brief, a Matrigel (BD Biosciences) plug supplemented with 50 µg/ml fibronectin was made on a transwell filter (catalog no. 3422; Corning Life Sciences). The cells were allowed to invade toward a gradient of 10% FBS and 10% Nu serum for 7 d. The cells were stained with 4 µM Calcein for 15 min and imaged at 5-µm steps to a total distance of 120 µm. ImageJ software was used to quantify the number of cells in every 5-µm step image from 5 µm to 100 µm. Images were analyzed as previously described (Jacob et al., 2016). For quantification, at least 20 cells from three different fields per treatment were counted.

### Migration assays and time-lapse analyses

For scratch assays, wells of a 96-well dish were coated with rat tail collagen (catalog no. 354249; Corning Life Sciences) and allowed to set for 1 h at room temperature. Wells were then washed once with PBS, and cells were plated to produce a confluent monolayer 24 h later and serum starved overnight. A WoundMake (Essen BioScience) was used to make scratches across each well. Wells were then rinsed twice with PBS and plated in low-serum media (DMEM + 2% FBS). Images were acquired over the center of the scratch every 2 h for 48 h. IncuCyteZOOM software was used to measure percent wound closure over time.

Chemotaxis assays were performed with the IncuCyte Live-Cell Analysis System according to manufacturer protocol. In brief, 1,000 subconfluent, serum-starved cells were plated into either fibronectin-coated or collagen-coated upper wells of a 96-well plate (IncuCyte ClearView 96-well Chemotaxis Plate, catalog no. 458). Upper chambers were supplied either regular growth serum (DMEM + 10% FBS) or low serum (2% FBS). The top and bottom wells were imaged every 2 h for 48 h. IncuCyte analysis software was used to analyze and quantify changes in cell area over time, normalized to the initial top chamber seeded cell count.

For individual cell time-lapse migration analysis, cells were plated on collagen-coated 35-mm glass-bottom dishes. Cells were serum starved overnight in media supplemented with SiR-actin. Cells were then washed once with PBS supplemented with DAPI at 1:20,000 and once again with PBS and then placed in full growth media supplemented with SiR-actin. Cell dishes were then placed in a climate-controlled chamber and images taken every 10 min. Images were taken on either a Zeiss LSM 880 with Zen Blue software (Zeiss) or with a Nikon A1R confocal system with NIS Elements software (Nikon) in a live-imaging chamber. Individual cell migration dynamics for individual cells was analyzed using ImageJ and the Manual Tracking plugin (Fabrice Cordelires, Institut Curie, Orsay, France). Generated values were then used to determine MSD, velocity, and directionality as described previously (Gorelik and Gautreau, 2014).

For time-lapse analysis of FA sites, cells were transfected with GFP-paxillin with jetPRIME (Polyplus) according to the manufacturer’s recommendations. Cells were then seeded onto a collagen-coated glass-bottom dish and incubated overnight with SiR-actin in minimal media. 1 h before imaging, cells were washed once with PBS and incubated in full growth media supplemented with SiR-actin. Cell dishes were then placed in a climate-controlled chamber and imaged every 4 min on Zeiss LSM 880. Images were preprocessed in ImageJ and uploaded to the FAAS server (https://faas.bme.unc.edu/; Berginski and Gomez, 2013) to measure adhesion dynamic characteristics. Adhesions tracked less than three consecutive frames were discarded. Life span was determined only for adhesions whose entire lifetime occurred during imaging.

To determine lamellipodia dynamics, MDA-MB-231 cells (control, FLAG-Rab40-3KO, and FLAG-Rab40b-4A) were plated on a collagen-coated glass-bottom dish and incubated overnight. Cell dishes were then placed in a climate-controlled chamber at 37°C and imaged by DIC using time-lapse microscopy. In all cases, 50 images with a time lapse of 3 s were taken for each cell. Images were taken on a Zeiss LSM 880 inverted microscope with Zen Blue software (Zeiss). To determine lamellipodia dynamics, a single pixel was chosen on the edge of the lamellipodia, and the DIC signal was measured at every time point. The maximum intensity of the DIC signal was than calculated and plotted along the x axis.

### **FLAG-**Rab40b IP and proteomic analyses

Putative Rab40b-binding proteins were identified by coimmunoprecipitation using anti-FLAG antibody–coated beads as described previously (Prekeris et al., 2000). Briefly, 100 µg affinity purified anti-FLAG antibody was bound to 100 µl Protein G–Sepharose beads. Antibodies were then cross-linked to beads using dimethyl pimelimidate dihydrochloride. Anti-FLAG antibody beads were then incubated with 2 ml of 1 mg/ml Triton X-100 cellular lysates (PBS, 1% Triton X-100, and 10 mM PMSF), followed by a wash with 5 ml PBS. Proteins were eluted from anti-FLAG antibody beads with 1% SDS and then analyzed using tandem mass spectrometry analysis as follows. Protein samples were digested with trypsin using the filter-aided sample preparation protocol (Wiśniewski et al., 2009). Liquid chromatographic analysis was performed in a Waters Acuity ultra-performance LC system (Waters Corporation). Peptide separation was performed on an ACQUITY UPLC HSS T3 250-mm analytical column. Data were acquired using Synapt G2 HDMS mass spectrometer and Masslynx 4.1 software (Waters Corporation) in positive-ion mode using data-independent acquisition Raw data were lock mass corrected using the doubly charged ion of [Glu1]-fibrinopeptide B (*m/z* 785.8426; [M+2H]2+).

Raw data files were processed and searched using ProteinLynx Global SERVER (PLGS) version 3.0.1 (Waters Corporation). Data were analyzed using trypsin as the cleavage protease, and one missed cleavage was allowed; fixed modification was set to carbamidomethylation of cysteines, and variable modification was set to oxidation of methionine. Minimum identification criteria included two fragment ions per peptide, five fragment ions per protein, and a minimum of two peptides per protein. The following parameters were used to generate peak lists: (1) minimum intensity for precursors was set to 135 counts, (2) minimum intensity for fragment ions was set to 25 counts, and (3) intensity was set to 750 counts. The UniProtKB/SwissProt human database was used for protein identification.

All candidate proteins identified in mass spectrometry were then filtered using two criteria: (1) candidate proteins had to be enriched at least 10-fold over IgG control; and (2) all RNA, DNA-binding proteins, and mitochondria proteins were eliminated as putative contaminants. Finally, only proteins enriched more than twofold in FLAG-Rab40b-4A samples were considered as putative Rab40b–Cullin5 substrate proteins (Fig. 1 B). The full list can be found in Table S1.

### **FLAG**-Rab40b-4A proteomics

In brief, WT FLAG-Rab40b or FLAG-Rab40b-4A were N-terminally FLAG tagged, expressed ubiquitously in MDA-MB-231 breast cancer cells, and immunoprecipitated using FLAG beads before mass spectrometry analysis. To determine Rab40b-interacting proteins (both WT and 4A), we established the following criteria. First, only proteins more than threefold IgG control (spectral counts) were analyzed. Second, any hits identified as nonspecific based on the CRAPome database were dismissed, as well as any additional DNA, RNA, and mitochondrial proteins. Finally, a twofold enrichment cutoff (spectral counts) was used to identify proteins preferentially bound to FLAG-Rab40b-4A versus FLAG-Rab40b. After analyzing protein hits from two independent experiments, we identified a set of Rab40b-binding proteins that were enriched in FLAG-Rab40b-4A compared with WT Rab40b. From the first run, 43.1% (81/188) of proteins were enriched in 4A versus WT. In the second run, 52.5% (32/61) of proteins were enriched in 4A versus WT. Proteins listed in Fig. 5 A are a shortened list of putative substrates, highlighting the theme of actin regulators. The full list can be found in Table S1.

### RT-PCR and quantitative PCR (qPCR)

Total RNA was extracted from 2 × 10^7^ MDA-MB-231 cells using TRIzol (Invitrogen) according to the manufacturer’s protocol. Reverse transcription to cDNA was performed with SuperScript III (Invitrogen) using random hexamer primers. PCR was performed using Taq polymerase (Invitrogen). To quantify the percent knockdown, cDNA from mock- or siRNA-treated cells was analyzed in triplicate by qPCR amplification using Sybr Green qPCR Master Mix using Applied Biosystems ViiA7 Real-Time PCR System. The qPCR amplification conditions were 50°C (2 min), 95°C (10 min), 40 cycles at 95°C (15 s), and 60°C (1 min). Primer pairs were designed to amplify mRNA-specific fragments, and unique products were tested by melt-curve analysis. Amplification efficiency was calculated using the slope of the regression line in the standard curve. Targets were normalized to GAPDH. The following primers used for qPCR were from PrimerBank (https://pga.mgh.harvard.edu/primerbank/): RAB40A forward (5′-CTG​CGG​CAC​AGG​ATG​AAT​TG-3′), RAB40A reverse (5′-AGG​CTG​CTC​TTG​TGA​GTG​GA-3′), RAB40B- forward (5′-GTC​CGG​GCC​TAC​GAC​TTT​C-3′), RAB40B reverse (5′-GGC​CTG​AAG​TAT​CCC​AGA​GC-3′), RAB40C forward (5′-GGC​CCA​ACC​GAG​TGT​TCA​G-3′), and RAB40C reverse (5′-GGA​CTT​GGA​CCT​CTT​GAG​GC-3′).

### Zymography assay

Control, FLAG-Rab40b, and FLAG-Rab40b-4A MDA-MB-231 cells were incubated in the complete medium at 37°C. After 24 h of incubation, the medium was replaced with Opti-MEM (Invitrogen), and cells were incubated at 37°C for another 24 h. Cell medium was collected and briefly centrifuged to clear the lysate. Equal concentrations of each sample were loaded into a 7.5% acrylamide gel supplemented with gelatin type A from porcine skin. After running the gel, gels were washed with water and incubated twice in 2.5% Triton X-100 for 30 min. Gels were then incubated in substrate buffer (50 mM Tris, pH 8.0, and 5 mM CaCl_2_) for 48 h at 37°C. Gels were stained with Coomassie for 1 h, destained, and imaged on Gel Doc EZ Imager (Bio-Rad Laboratories). MMP2/9 secretion was quantified using Quantity One Software (Bio-Rad Laboratories).

### siRNA knockdown

For EPLIN, siRNAs were purchased from Dharmacon. D001210-01-05 (nontargeting), D010663-02-0002 (LIMA1 #1), and D010663-03-0002 (LIMA1 #2) siRNAs were transfected using jetPRIME according to manufacturer protocol. Cells were trypsinized and replated onto collagen-coated coverslips at 24 h. Cells were fixed at 48 h, stained, and imaged with a Nikon A1R. For Rab40b and Rab40c, siRNAs were purchased from Sigma. Mission siRNA universal negative control (SIC001; Sigma), Rab40b siRNA (SASI_Hs01_00150368), and Rab40c siRNA (SASI_Hs01_00031938) were transfected using Lipofectamine RNAiMAX (Invitrogen) according to manufacturer protocol.

### IF microscopy

MDA-MB-231 cells were seeded onto collagen-coated glass coverslips and grown in full growth media unless otherwise noted for at least 24 h. Cells were washed with PBS and fixed in 4% paraformaldehyde for 15 min. Samples were then washed three times in PBS then incubated in blocking serum (1× PBS, 5% normal donkey serum, and 0.3% Triton X-100) for 1 h at room temperature. Primary antibodies were then diluted at 1:100 in dilution buffer (1× PBS, 1% BSA, and 0.3% Triton X-100) overnight at 4°C. Samples were then washed three times with PBS and incubated with fluorophore-conjugated secondary antibodies (1:100 in dilution buffer) for 1 h at room temperature. Cells were then washed three times in PBS and mounted onto glass slides. Cells were then imaged on either an inverted Zeiss Axiovert 200M deconvolution microscope with a 63× oil-immersion lens and Sensicam QE charge-coupled device camera or a Nikon A1R. Z-stack images were taken at a step size of 100–500 nm.

### CRISPR-Cas9 knockout lines

Rab40 CRISPR 3KO cell lines were generated using MDA-MB-231 cell line stably expressing tetracycline-inducible Cas9. For Rab40a, guide RNAs targeting 5′-CCT​CAA​AGA​AGG​TCA​CAC​CC-3′ (exon 3) and 5′-TTG​GCT​CGG​GAG​GCC​GAG​CA-3′ (exon 3) were transfected into doxycycline (Dox)-induced Cas9 MDA-MB-231s. For Rab40b, guide RNAs targeting 5′-TCC​AGG​GAT​ACT​TCA​GGC​CA-3′ (exon 3) and 5′-TCT​GGC​GGC​CGA​GCA​AGG​GT-3′ (exon 5) were transfected into Dox-induced Cas9 MDA-MB-231s. For Rab40c, guide RNAs targeting 5′-TAC​CGT​TAC​TGT​AGG​CGT​AC-3′ (exon 1) and 5′-AGG​TAG​TCG​TAG​CTC​TTC​AC-3′ (exon 3) were transfected into Dox-induced Cas9 MDA-MB-231s. Cells were split 24 h after transfection and seeded for single colonies. Clones were screened by PCR for followed by cloning and genotyping. First, Rab40b and Rab40c double knockout was generated. One of the Rab40b/c double-knockout clones were than used to generate Rab40-3KO. Two different triple-knockout lines were then used for experiments.The mutations in all Rab40a, Rab40b, and Rab40c alleles are as follows (asterisks mark where the mutant allele sequence diverges from WT due to a frameshift mutation): Rab40c allele (both alleles have the same mutations), MGSQGSPVKSYDYLLKFLLVGDSDVGKGEILESLQDGAAESPP*TVTGSTTRPPPSCWTAGA; Rab40b allele #1, MSALGSPVRAYDFLLKFLLVGDSDVGKGGEILASLQDGAAESPYGHPAGIDYKTTTILLDGRRVKLQLWDTS*AREDFVPYSAPTPGAHRV; Rab40b allele #2, MSALGSPVRAYDFLLKFLLVGDSDVGKGGEILASLQDGAAESPYGHPAGIDYKTTTILLDGRRVKLQLWDTSGQGRFCTIFRSYSRGAQGVILVYDIANRWSFDGIDRWIKEIDEHAPGVPKILVGNRLHLAFKRQVPTEQAQAYAERLGVTFFEVSPLCNFNITESFTELARIVLLRHGMDRLWRPSK*C; Rab40c allele #1 (Rab40-3KO-1 line), MSAPGSPDQAYDFLLKFLLVGDRDVGKSEILESLQDGAAESPYSHLGGIDYKTTTILLDGQRVKLKLWDTSGQGRFCTIFRSYSRGAQGVILVYDIANRWSFEGMDRWIKKIEEHAPGVPKILVGNRLHLAFKRQVPREQAQAYAERLGVTFFEVSPLCN*FNIIESFTELARIVLLRHRMNWLRPSKVLSLQDLCCRTIVSCTPVHLVDKLPLPSTLRSHLKSFSMAKGLNARMMRGLSYSLTTSSTHKSSLCKVEIVCPPQSPPKNCTRNSCKIS; Rab40c allele #2 (Rab40-3KO-1 line), MSAPGSPDQAYDFLLKFLLVGDRDVGKSEILESLQDGAAESPYSHLGGIDYKTTTILLDGQRVKLKLWDTSGQGRFCTIFRSYSRGAQGVILVYDIANRWSFEGMDRWIKKIEEHAPGVPKILVGNRLHLAFKRQVPREQAQAYAERLG*GGRARY; Rab40c allele #1 (Rab40-3KO-2 line), MSAPGSPDQAYDFLLKFLLVGDRDVGKSEILESLQDGAAESPYSHLGGIDYKTTTILLDGQRVKLKLWDTSGQGRFCTIFRSYSRGAQGVILVYDIANRWSFEGMDRWIKKIEEHAPGVPKILVGNRLHLAFKRQVPREQAQAYAERL*GVTFFEVSPLCNFNIIESFTELARIVLLRHRMNWLRPSKVLSLQDLCCRTIVSCTPVHLVDKLPLPSTLRSHLKSFSMAKGLNARMMRGLSYSLTTSSTHKSSLCKVEIVCPPQSPPKNCTRNSCKIS; and Rab40c allele #2 (Rab40-3KO-2 line), MSAPGSPDQAYDFLLKFLLVGDRDVGKSEILESLQDGAAESPYSHLGGIDYKTTTILLDGQRVKLKLWDTSGQGRFCTIFRSYSRGAQGVILVYDIANRWSFEGMDRWIKKIEEHAPGVPKILVGNRLHLAFKRQVPREQAQAYAERL*premature STOP codon.

### Glutathione bead pull-down and IP assays

Glutathione bead pull-down assays were performed as described previously (Mangan et al., 2016). Briefly, glutathione beads (GoldBio) were coated with GST-Cullin5 or GST control in PBS and incubated with MDA-MB-231 control or Rab40b mutant cell lysates (PBS, 1% Triton X-100, and 10 mM PMSF) in a final volume of 0.5 ml of reaction buffer (50 mM Hepes, pH 7.4, 150 mM NaCl, 0.1% Triton X-100, and 1 mM PMSF). Samples were incubated at 25°C for 1 h while rotating, pelleted by centrifugation at 2,000 *g* for 3 min, and rinsed with reaction buffer (1 ml × 3), and bound proteins were eluted with 1% SDS, analyzed by SDS–PAGE, and stained with Coomassie blue or immunoblotted and scanned using a LI-COR Odyssey scanner (LI-COR Biosciences).

For EPLIN and FLAG-Rab40b coimmunoprecipitation assays, 1 ml of 1 mg/ml of cell lysates was precleared with 25 μl of Protein G–Sepharose beads for 1 h at 4°C and then incubated with 5 µg of IgG control (115–006-006; Jackson Laboratory) or anti-FLAG antibody (16639–1-AP; Protein Tech) and incubated at 37°C while rotating. After 60 min, 50 µl of Protein G beads was added and samples were incubated at 37°C for 60 min while rotating. Samples were then washed, pelleted by centrifugation at 2,000 *g* for 3 min, and washed four times in wash buffer (50 mM Hepes, pH 7.4, 300 mM NaCl, 0.1% Triton X-100, and 0.1% SDS). Bound proteins were eluted with 1% SDS, analyzed by SDS–PAGE, immunoblotted, and scanned using a LI-COR Odyssey scanner.

### EPLIN ubiquitylation assay

293t cells were plated into 10-cm dishes. When cells were 50% confluent, cells were transfected with siRNAs listed above (control, Rab40b, Rab40c, or Rab40b and Rab40c). After 24 h, were transfected with FLAG-α-EPLIN using Lipofectamine 2000 (Invitrogen). After 24 h, cells were treated with 10 µM MG132 overnight then lysed in Tris-buffer containing 10 mM Iodoacetamide. Cell lysates were subject to IP with FLAG antibody (M2; Sigma) followed by WB with FLAG or ubiquitin antibody (P4D1; Santa Cruz Biotechnology).

### Image analysis

#### Analysis of FA number and size

FA quantification performed in ImageJ and was adapted from Horzum et al. (2014). In brief, maximum intensity projections for relevant z-planes were created, and images were loaded into ImageJ. Background was minimized using the Subtract Background and EXP tools, images were filtered using the Log3D/Mexican Hat plugin, and thresholds were then applied manually (method = default). Individual cells were defined by hand, and FAs were determined with the “Analyze Particle” command. Resulting particle outlines were then compared with the original image to ensure fidelity of the analysis. For live-imaging analysis of FA, images were processed in ImageJ and uploaded to the FAAS server (Berginski and Gomez, 2013).

#### Analysis of FAs positive for zyxin

To determine colocalization of zyxin-positive FAs, zyxin/paxillin dual-stained images were first split using the “Split Channels” tool in ImageJ, background was subtracted, and thresholds were adjusted as above. Individual cells were individually defined manually and then analyzed with the colocalization plugin (Pierre Bourdoncle, Institut Jacques Monod, Service Imagerie, Paris, France) to determine zyxin-positive paxillin patches. Determination of cortactin-positive actin puncta was done similar to that of zyxin-positive FAs.

#### Quantification of cellular EPLIN and nonmuscle Myosin IIA/B

To measure the levels of EPLIN and nonmuscle Myosin IIA/B associated with actin cytoskeleton, MDA-MB-231 cells (control, Rab40-3KO, and FLAG-Rab40b-4A) were fixed and stained with phalloidin–Alexa Fluor 594 and either EPLIN or nonmuscle Myosin IIA/B antibodies. Randomly selected cells from two random fields were then imaged using the same exposure time. Masks were generated for each cell, and sum-fluorescence intensity of EPLIN or nonmuscle Myosin IIA/B was calculated for each cell. The sum intensity was then expressed as fluorescence per 1 µm^2^ for each cell analyzed. The experiment was repeated three times and the values expressed as means and SDs calculated from all cells analyzed.

#### Cell spreading analysis

To determine the effect of Rab40b on cell–ECM adhesion, MDA-MB-231 cells (control, Rab40-3KO, and FLAG-Rab40b-4A) were plated on collagen-coated coverslips and incubated for 90 min. Cells were then fixed and stained with phalloidin–Alexa Fluor 594. Randomly selected cells from three random fields were then imaged. Masks were generated for each cell, and the area covered by each individual cell was calculated. The experiment was repeated three times and the values expressed as means and SDs calculated from all cells analyzed.

#### Line-scan analysis of EPLIN distribution in lamellipodia

To determine the subcellular distribution of EPLIN, MDA-MB-231 cells (control, Rab40-3KO, and FLAG-Rab40b-4A) were plated on collagen-coated coverslips and incubated for 24 h. Cells were then fixed and stained with anti-EPLIN antibodies and phalloidin–Alexa Fluor 594. For individual isoforms, cells were plated on collagen-coated coverslips and incubated overnight, transfected with GFP-EPLIN-α or EPLIN-β, and then incubated for 24 h. Cells were then fixed and stained with anti-EPLIN antibodies and phalloidin–Alexa Fluor 594. Z-stack images were acquired, and maximum projection intensities were created of each image. Only cells with well-defined lamellipodia (as determined by imaging phalloidin–Alexa Fluor 594) were chosen for this analysis. Lines were then drawn across the lamellipodia perpendicular to nucleus. The intensity of EPLIN and F-actin in each pixel along this line was determined with either ImageJ or 3i Slidebook imaging software. Intensities along each line were normalized, and the front edge of either actin or EPLIN was determined where intensity was at least 20% of maximum.

### Statistical analysis

Statistical analysis for all experiments was determined using GraphPad Prism Software (GraphPad). A two-tailed Student's *t* test was used to determine statistical significance unless otherwise noted. Data were collected from at least three independent experiments unless otherwise noted. In all cases, P ≤ 0.05 was regarded as significant. Error bars represent standard errors unless otherwise noted. For all IF experiments, at least five randomly chosen image fields per condition were used for data collection. For quantitative IF analysis, the same exposure was used for all images in that experiment and quantified using ImageJ.

### Online supplemental material

**Figure S6.**
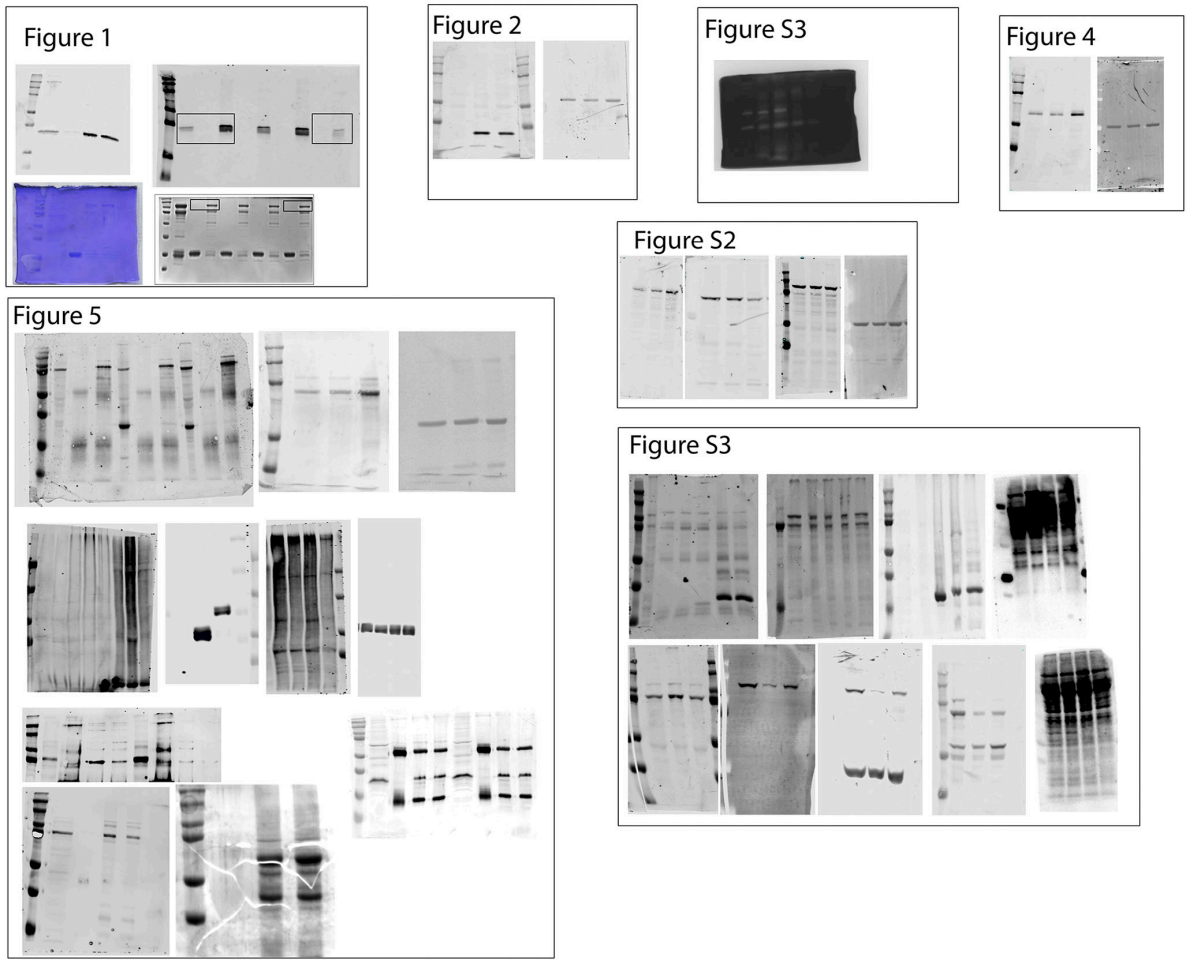
**Uncropped WB images shown in previous figures.**

Fig. S1 shows some of the data from cell migration analysis. Fig. S2 provides additional data on FA maturation. Fig. S3 provides additional evidence for Rab40b/CRL5 roles in regulating actin dynamics and MMP secretion. Fig. S4 shows the effects of Rab40-3KO on nonmuscle Myosin IIA/B localization. Fig. S5 provides data on the involvement of Rab40/CRL5 in regulating cell spreading and lamellipodia dynamics. Fig. S6 shows all uncropped WB images. Videos 1 and 2 show cell migration in control and Rab40b-4A–expressing cells. Videos 3 and 4 show GFP-paxillin–labeled FA dynamics in control and Rab40b-4A–expressing cells. Videos 5, 6, and 7 show lamellipodia dynamics in control, Rab40-3KO–, and Rab40b-4A– expressing cells. Table S1 shows full results of mass spectrometry experiments.

## Supplementary Material

Table S1 lists the full results of mass spectrometry experiments.
